# A Comprehensive Bioinformatic Analysis of RNA-seq Datasets Reveals a Differential and Variable Expression of Wildtype and Variant UGT1A Transcripts in Human Tissues and Their Deregulation in Cancers

**DOI:** 10.3390/cancers16020353

**Published:** 2024-01-13

**Authors:** Dong Gui Hu, Shashikanth Marri, Julie-Ann Hulin, Ross A. McKinnon, Peter I. Mackenzie, Robyn Meech

**Affiliations:** College of Medicine and Public Health, Flinders Health and Medical Research Institute, Flinders University, Bedford Park, Adelaide 5042, Australia; shashikanth.marri@flinders.edu.au (S.M.); julieann.hulin@flinders.edu.au (J.-A.H.); ross.mckinnon@flinders.edu.au (R.A.M.); peter.mackenzie@flinders.edu.au (P.I.M.); robyn.meech@flinders.edu.au (R.M.)

**Keywords:** glucuronosyltransferase, cancer, alternative splicing, differential expression, deregulation, drug metabolism

## Abstract

**Simple Summary:**

UGT enzymes metabolize and detoxify numerous small molecules that are important in cancer, including carcinogens, endogenous growth regulators, and anticancer drugs. Alternatively spliced UGT transcripts can encode truncated proteins that inhibit canonical UGTs, thus reducing detoxification activity. We assessed the expression of specific variant transcripts, designated as UGT1A_v2 and _v3, in six different cancers using RNA-seq datasets with large cohorts of paired normal and tumor tissues. Our results show high interindividual variation in v2 and v3 transcript abundance, as well as tissue- and tumor-specific expression patterns. These findings suggest that the variants have tissue-specific impacts on glucuronidation and may have a more significant role in tumors than in normal tissues. The high interindividual variability is likely relevant to differing personalized drug metabolisms through the UGT conjugation pathway. Finally, our discovery of novel UGT1A variant transcripts further highlights the diversity of the UGT1A transcriptome and proteome.

**Abstract:**

The *UGT1A* locus generates over 60 different alternatively spliced transcripts and 30 circular RNAs. To date, v2 and v3 transcripts are the only variant UGT1A transcripts that have been functionally characterized. Both v2 and v3 transcripts encode the same inactive variant UGT1A proteins (i2s) that can negatively regulate glucuronidation activity and influence cancer cell metabolism. However, the abundance and interindividual variability in the expression of v2 and v3 transcripts in human tissues and their potential deregulation in cancers have not been comprehensively assessed. To address this knowledge gap, we quantified the expression levels of v1, v2, and v3 transcripts using RNA-seq datasets with large cohorts of normal tissues and paired normal and tumor tissues from patients with six different cancer types (liver, kidney, colon, stomach, esophagus, and bladder cancer). We found that v2 and v3 abundance varied significantly between different tissue types, and that interindividual variation was also high within the same tissue type. Moreover, the ratio of v2 to v3 variants varied between tissues, implying their differential regulation. Our results showed higher v2 abundance in gastrointestinal tissues than liver and kidney tissues, suggesting a more significant negative regulation of glucuronidation by i2 proteins in gastrointestinal tissues than in liver and kidney tissues. We further showed differential deregulation of wildtype (v1) and variant transcripts (v2, v3) in cancers that generally increased the v2/v1 and/or v3/v1 expression ratios in tumors compared to normal tissues, indicating a more significant role of the variants in tumors. Finally, we report ten novel UGT1A transcripts with novel 3′ terminal exons, most of which encode variant proteins with a similar structure to UGT1A_i2 proteins. These findings further emphasize the diversity of the UGT1A transcriptome and proteome.

## 1. Introduction

The human UDP-glucuronosyltransferase family (UGT) comprises nine UGT1 (1A1 and 1A3–1A10) and ten UGT2 (2A1, 2A2, 2A3, 2B4, 2B7, 2B10, 2B11, 2B15, 2B17, and 2B28) enzymes [1]. These UGTs convert numerous endogenous and exogenous small lipophilic compounds (e.g., steroid hormones, bile acids, bilirubin, fatty acids, therapeutic drugs, carcinogens, dietary constituents, and environmental toxins) into water-soluble glucuronide conjugates, thus promoting their excretion from the body [2]. UGTs have an important role in maintaining the homeostasis of signaling molecules, protecting against toxicity, and governing drug efficacy [1,2]. Variation in UGT activity can have a significant clinical impact. For example, UGT1A1-mediated glucuronidation is critical for the excretion of bilirubin (a major metabolite of heme) and SN-38 (the active metabolite of the anticancer drug Irinotecan) [3,4,5]. Patients carrying the low-activity UGT1A1*28 allele display congenital hyperbilirubinemia (e.g., Crigler–Najjar and Gilbert syndromes) [6,7,8,9,10] and irinotecan toxicities such as neutropenia and myelosuppression [3,11,12,13,14].

Glucuronidation capacity is also determined by the level of *UGT* gene expression. *UGTs* are subject to dynamic regulation by a network of constitutive and inducible (e.g., ligand-dependent) transcription factors, leading to tissue-selective expression patterns, high interindividual variability, and the potential for dysregulation in disease states such as cancer [1,2]. Emerging work suggests that another important determinant of glucuronidation activity is the production of variant UGT proteins from alternatively spliced transcripts. For example, we and others have shown that truncated UGT variants can oligomerize with full-length UGT proteins and inhibit their functions [15,16].

**Figure 1:**
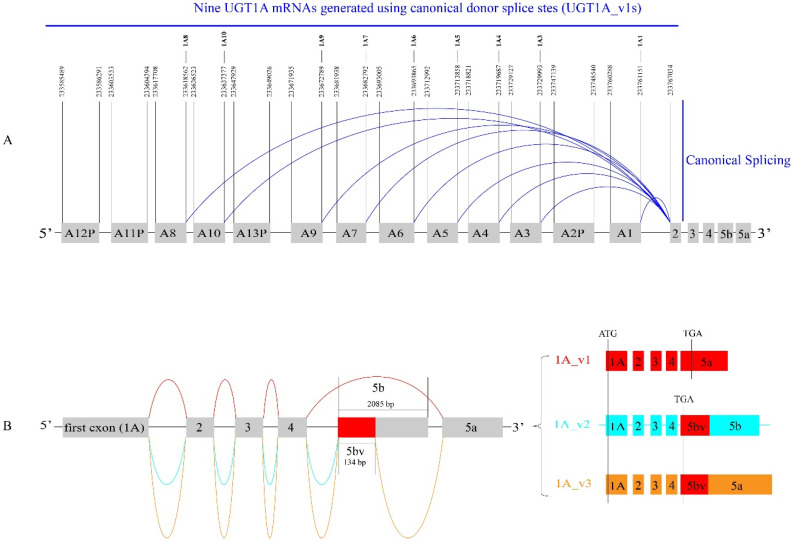
Canonical and variant splicing of the *UGT1A* locus generate wildtype and variant UGT1A transcripts. The *UGT1A* locus has nine different wildtype first exons (1, 3–10) and exons 2–5. The genomic positions of these canonical exons are annotated according to the human genome GRCH/hg38 assembly (**A**). Canonical splicing (highlighted in BLUE) where each individual first exon is ligated to downstream exons 2–5a generates nine wildtype UGT1A transcripts (v1s). Exon 5b (2085 bp) is an alternative exon that is located between exons 4 and 5a (**A**,**B**). Variant splicing (highlighted in AQUA) where exon 5b is ligated to exon 4 generates nine variant UGT1A transcripts (v2s) (**B**). The 5′ 134-bp fragment of exon 5b is termed exon 5bv. Variant splicing (highlighted in YELLOW) of 5bv between exons 4 and 5a generates nine variant UGT1A transcripts (v3s).

The nine UGT1A enzymes are encoded by a single gene locus on chromosome 2q37.1 [1]. The *UGT1A* locus comprises nine functional first exons (*1A1*, *1A3–1A10*), four pseudo-first exons (*1A2P*, *1A11P*, *1A12P*, *1A13P*), and four common exons (i.e., 2, 3, 4, 5a) (Figure 1A). Each first exon is transcribed from a separate promoter generating a pre-mRNA that contains any downstream first exons as well as the four common exons. To produce each canonical UGT1A mRNA, the most distal (5′) first exon is spliced to exon 2, excising any intervening first exons. There are nine such canonical mRNAs, designated as UGT1A_v1s (1A1, 1A3–1A10), that each contain a unique first exon followed by the four common exons 2, 3, 4, and 5a (Figure 1B). The *UGT1A* locus also generates 65 alternatively spliced transcripts and 34 circular RNAs, highlighting the complexity of the UGT1A transcriptome [15,16,17]. Alternate transcripts typically result from the use of alternative 5′ or 3′ splice sites, from exon skipping, or from the inclusion of alternative exons. In particular, intron 4 contains two overlapping alternative exons, designated as exon 5b (2086 bp) and exon 5bv (134 bp) [15,18,19]. Exon 5bv contains the 5′ 134-bp region of exon 5b [15]. Alternative splicing of exons 5b, 5bv, and the canonical exon 5a generates a total of 18 alternate transcripts. The nine UGT1A_v2 alternate transcripts are comprised of exons 1, 2, 3, 4, and 5b. The nine UGT1A_v3 transcripts are comprised of exons 1, 2, 3, 4, 5bv, and 5a (Figure 1B).

Full-length UGT1A proteins, named isoforms_1 (UGT1A_i1s), are encoded by the canonical UGT1A_v1 transcripts. UGT1A_i1 proteins comprise, in order, an N-terminal signal peptide, a substrate-binding domain, a co-substrate-binding domain, a transmembrane (TM) region, and a C-terminal cytoplasmic tail [1]. The C-terminal 99 aa are encoded by exon 5a [2]. In contrast, the v2 and v3 transcripts (which contain exon 5b and 5bv/5a, respectively) encode truncated UGT proteins that are designated as isoforms-2 (UGT1A_i2) (Figure 1B). Because exon 5b and exon 5bv share a common 5′ sequence, the v2 and v3 transcripts have the same Open Reading Frame (ORF) and hence encode the same set of UGT1A_i2 proteins. These truncated proteins lack the last 99-aa and instead contain a novel 10-aa C-terminal peptide (RKKQQSGRQM) [18,19]. As shown in Figure 1B, v2 and v3 transcripts contain different 3′ untranslated regions (3′UTR), and thus, they may be subject to different post-transcriptional regulation, including regulation by miRNAs and RNA-binding proteins.

Out of the nearly 100 reported alternatively spliced and circular UGT1A transcripts, v1 and v2 transcripts are the only ones known to be translated into proteins (i.e., UGT1A_i2) in human tissues and cell lines [17,18,19]. UGT1A_i2 proteins lack transferase activity, but they can interact with _i1 proteins to inhibit their activity, indicative of dominant-negative regulation [20,21,22,23]. Thus, the i2/i1 expression ratio is considered an important determinant of glucuronidation activity [24]. In addition, i2 proteins were found to interact with non-UGT proteins (e.g., pyruvate kinase) to modulate cancer cell metabolism [25,26]. Previous work examined the abundance of v2 and v3 transcripts (v2/v3) together in small cohorts of normal or cancerous tissues including drug-metabolizing tissues (liver, kidney, intestine, colon) [18,19,23,27,28,29]. Results from these studies have reported that the expressed ratio of v2/v3 versus v1 transcripts is generally less than 10% in normal liver tissues [27,28,29,30] but relatively higher in normal kidney (16%) and kidney tumor tissues (22%) [28]. The limitations of these studies are that they only analyzed limited numbers of normal and tumor samples and did not quantify v2 and v3 transcripts separately. Therefore, the expression profiles of v2 and v3 transcripts in normal and cancerous human tissues and their potential deregulation in cancer remains unknown.

In the present study, we comprehensively assessed the expression levels and interindividual variability of the three types of UGT1A transcripts (v1, v2, and v3) in human tissues and their potential deregulation in cancers using RNA-seq datasets with large cohorts of normal tissues or paired normal and tumor tissues from patients with six different cancer types (liver, kidney, esophagus, stomach, colon, and bladder cancer). Furthermore, we report the discovery of ten novel types of UGT1A transcripts that contain a novel 3′ terminal exon.

## 2. Materials and Methods

### 2.1. Analysis of Differential and Variable Expression of Known UGT1A Transcripts in Normal Human Tissues and Their Deregulation in Cancers

The Sequence Read Archive (SRA) is the largest publicly available repository of high-throughput sequencing data, including RNA-seq datasets from human normal and tumor tissues (https://www.ncbi.nlm.nih.gov/sra) (accessed on 1 July 2023). As shown in Figure 1, the *UGT1A* locus generates three sets of transcripts (i.e., v1, v2, v3) that each comprises nine different isoforms (1A1, 1A3-10). In this study, we quantified the total expression level of each set of nine transcripts using set-specific probe sequences (30 nt) (Table S1). Briefly, v1 transcripts were quantified using probe v1 (5′-CATCAATGACAAAAG/TTACAAGGAGAACAT-3′) that spanned the E4/E5a splice junction, and v3 transcripts were quantified using probe v3 (5′-CTTCCCACCTTTGA/GTTACAAGGAGAACAT-3′) that spanned the E5bv/E5a splice junction. v2 transcripts lack a specific splice junction for quantification; however, both v2 and v3 transcripts have the specific E4/E5bv splice junction. Using probe v2/v3 (5′-CATCAATGACAAAAGAAAG/AAGCAGCAGTC-3′) spanning this common splice junction, v2 and v3 transcripts (v2/v3) were quantified together. The number of v2 sequence reads was thus calculated by deducting the number of v3 sequence reads from the number of v2/v3 sequence reads.

Using the above-described transcript-specific probes, we assessed the expression of three sets of UGT1A transcripts (v1, v2, v3) using the HPA (Human Protein Atlas) RNA-seq datasets, generated from 27 different types of normal human tissues (ERP003613) [31]. Each HPA tissue set contains 4–13 samples from different individuals (Supplemental Table S4). To assess potential deregulation in human cancers, we analyzed six RNA-seq datasets from paired normal and tumor tissues that were generated from large cohorts of patients with different cancers, namely, hepatocellular carcinoma (SRP401130) [32], clear cell renal cell carcinoma (SRP238334) [33], colorectal cancer (SRP107326) [34], stomach cancer (SRP172499) [35], esophageal squamous cell carcinoma (SRP193095) [36], and urinary bladder cancer (SRP212702) [37] (Table 1 and Table S1–S7). Briefly, we downloaded the raw RNA sequencing files (fastq) of the above-described SRA datasets from the NCBI database using the SRA toolkit (https://github.com/ncbi/sra-tools/wiki/01.-Downloading-SRA-Toolkit) (accessed on 1 September 2023). The SRA toolkit provides a set of command-line utilities that facilitate the retrieval, manipulation, and analysis of data from the SRA database. The ‘prefetch’ and ‘fastq-dump’ SRA toolkit commands were used to download fastq from the SRA database. The Unix ‘grep’ command was used for searching and counting sequence reads in the downloaded fastq files that contained the exact matches for the transcript-specific probes. The number of sequence reads for all transcripts that were found in all samples of these SRA datasets are provided in Tables S1–S7. The expression level of a transcript in a sample was normalized using the number of the total sequence reads in the same sample and presented as normalized reads per 10^9^ total sequence reads.

### 2.2. Discovery and Quantification of Novel UGT1A Transcripts with 3′ Abnormalities

We also quantified the expression levels of transcripts v1, v2, and v3 using UGT-enriched CaptureSeq datasets that were generated from three different normal tissue types (i.e., liver, kidney, intestine/colon) and two different tumor tissue types (i.e., kidney and intestine/colon) [23,28]. Each CaptureSeq RNA sample was pooled from 3–5 individual samples. The UGT-enrichment factor was estimated to be approximately 1000-fold [16]. Previous analyses of these CaptureSeq datasets identified about 60 different variant UGT1A transcripts [16]. In the present study, we reanalyzed these CaptureSeq datasets with a focus on identifying splice junctions that ligated 1) any downstream sequences to the 3′ end of exon 4 or 2) any upstream sequences to the 5′ end of exon 5a or exon 5b using cryptic splice sites. Briefly, the UGT-CaptureSeq data (GSE80463) were downloaded from NCBI GEO, and the 100-nt paired-end reads were merged into a single 200-nt fragment using Illumina Paired-End reAd mergeR (PEAR) [38]. The merged reads were searched for sequences in which any downstream sequences were spliced to exon 4 (demarcated by 5′-ATTTAGAAAATGCTCTAAAAGCAGTCATCAATGACAAAAG-3′) or any upstream sequences were spliced to exon 5a (demarcated by 5′-TTACAAGGAGAACATCATGCGCCTCTCCAGCCTTCACA-3′) or exon 5b (demarcated by: 5′-AAAGAAGCAGCAGTCAGGAAGACAGATGTGAAGAGCTGGA-3′). This analysis identified 413, 4048, and 1025 unique sequences that were spliced to exons 4, 5a, and 5b, respectively (Tables S8–S10). We extracted a further 15-nt of downstream sequences from each of these reads and then aligned the sequences to definitively identify novel splice junctions. Using the SRA platform and transcript-specific probes, we obtained the sequence reads that contained the specific splice junctions of ten different types of novel UGT1A transcripts (v3down1, v3down2, v2/v3up, v2/v3down, vE5a1, vE5a2, vE5a3, vE2E5a, vE5c, vE5d) in all normal and tumor samples of the above-mentioned six different SRA datasets (Tables S2–S7).

### 2.3. Statistical Analysis 

The potential correlation between the expression levels of wildtype and variant UGT1A transcripts in normal or tumor tissues was assessed by Spearman ranking correlation analysis. The potential deregulation in the expression levels of wildtype or variant UGT1A transcripts in tumor tissues compared to matched normal tissues was assessed by Wilcoxon matched-pairs signed rank test. Both statistical analyses were conducted using GraphPad Prism (version 9.1.1) (GraphPad Software, San Diego, CA, USA). A *p* value of <0.05 was considered statistically significant.

## 3. Results 

### 3.1. Expression Profiles of UGT1A Transcripts in Normal Human Tissues

**Figure 2:**
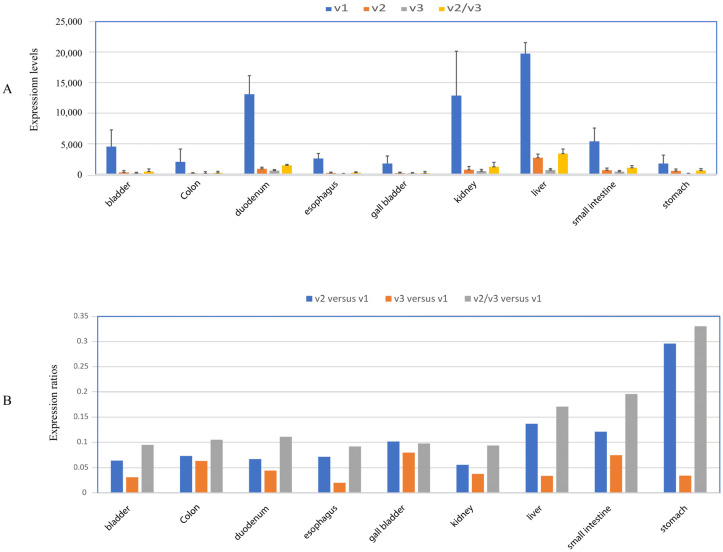
Expression profiles of UGT1A transcripts in human tissues. The RNA-seq dataset of the Human Protein Atlas (HPA) project was downloaded from the NCBI database, and the sequence reads of UGT1A transcripts were obtained using the SRA toolkit. Shown are the expression levels of UGT1A_v1, _v2, and _v3 transcripts in nine human tissues as indicated (**A**) and the expression ratios of v2 versus v1, v3 versus v1, and v2/v3 versus v1 (**B**) in these tissues.

To help guide the selection of tumor datasets in our subsequent analyses, we first assessed the distribution of UGT1A transcripts v1, v2, and v3 in 27 different normal human tissues using the human Protein Atlas (HPA) RNA-seq datasets [31]. UGT1A_v1 transcripts were found in sixteen tissues (adipose, appendix, bladder, colon, duodenum, esophagus, gall bladder, kidney, liver, prostate, salivary gland, skin, small intestine, stomach, testis, thyroid) (Figure S1). As shown in Figure 2A, v1 transcripts were considered abundant in nine tissues (bladder, colon, duodenum, esophagus, gall bladder, kidney, liver, small intestine, stomach), with the highest expression in liver tissue. All nine of these tissues also expressed v2 and v3 transcripts at levels that we considered to be low-to-moderate (ranging approximately from 10 to 30% of v1 levels) (Figure 2B). Tissues defined as having low levels of v1 transcripts generally had a very low or absent expression of v2 and v3 (Figure S1A). One intriguing exception to the generally low relative expression of v2 and v3 transcripts was testis, in which v2 transcript levels were 10 times higher than v1 transcripts (Figure S1B).

Because v2 and v3 transcripts both encode the same inhibitory _i2 proteins, the ratio of these variants to v1 transcripts can influence the glucuronidation capacity. We thus measured the ratio of v2 and v3 combined, relative to v1 (i.e., v2/v3 versus v1). This ratio was approximately 0.1 in bladder, colon, duodenum, esophagus, gall bladder, and kidney tissues; it was slightly higher (ranging from 0.17 to 0.32) in liver, small intestine, and stomach tissues (Figure 2B). We also compared the relative abundance of v2 to v3 transcripts (i.e., v2 versus v3) in each tissue. In colon, duodenum, small intestine, gall bladder, and kidney tissues, v2 and v3 transcripts showed similar abundance, with the v2 versus v3 ratio ranging from 1.1 to 1.5. However, in bladder, esophagus, liver, and stomach tissues, v2 was most abundant, with v2 versus v3 ratio ranging within 2.5–7. Guided by these tissue profiles and the availability of suitable RNA-seq datasets, we proceeded to measure the abundance of transcripts v1, v2, and v3 in large cohorts of paired normal and tumor specimens from patients with six different cancer types (i.e., liver, kidney, colon, stomach, esophagus, and bladder cancer).

### 3.2. Expression Profiles of UGT1A Transcripts in Normal Liver Tissues and Their Deregulation in Liver Tumor Tissues

The abundance of UGT1A transcripts v1, v2, and v3 was extracted from an RNA-seq dataset representing 65 paired hepatocellular carcinoma (HCC) tumor- and adjacent normal tissues [32]. Using these data, we first assessed interindividual variability in transcript abundance. All three types of transcripts were expressed in almost all normal- and tumor liver samples and showed high interindividual variability (Figure S2). v1 transcripts showed much greater expression variability within the tumor set than in normal tissues (1697- and 17-fold, respectively). v2 and v3 transcripts also showed greater expression variability within tumors than in normal tissues; however, the discrepancy was not as great as that observed for v1 (Table 1, Figure S2).

**Figure 3:**
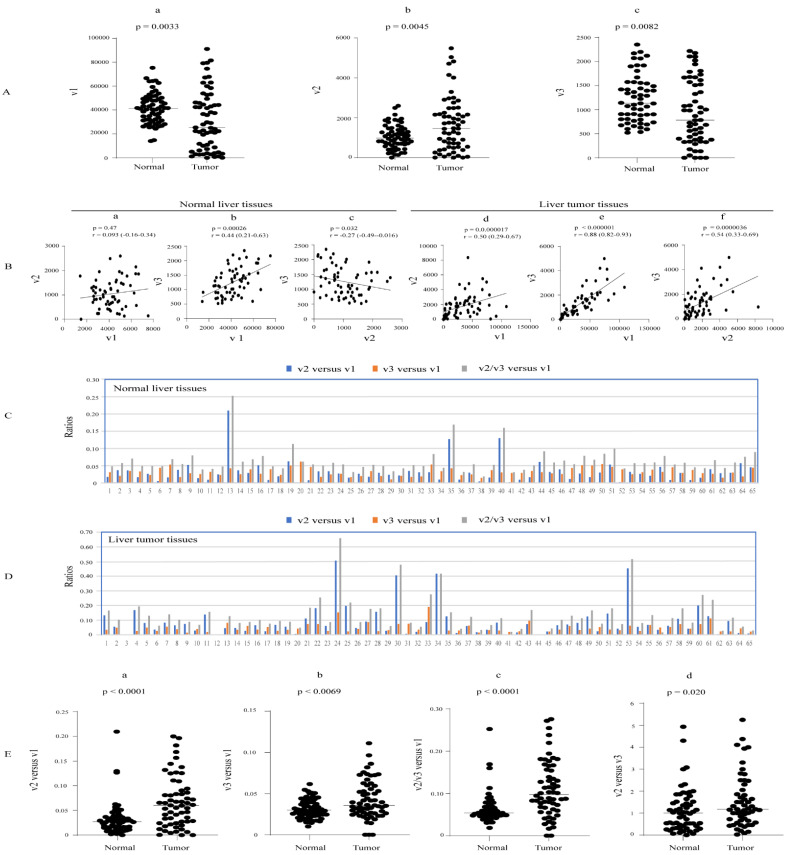
Expression of UGT1A transcripts in liver tumor and matched adjacent normal tissue. The RNA-seq dataset of 65 paired liver tumor and adjacent normal liver tissues (SRP401130) was downloaded from the NCBI database, and the sequence reads of UGT1A transcripts were obtained using the SRA toolkit. Shown are the expression levels of UGT1A transcripts v1, v2, and v3 (**A**) and analyses of their correlation (**B**) and relative abundance (**C**–**E**) in both normal and tumor tissues. Deregulation analysis using Wilcoxon matched-pairs signed rank test and correlation analysis using Spearman ranking test were conducted using GraphPad Prism (version 9.1.1). *p* < 0.05 is considered statistically significant.

We next examined whether the transcripts were up- or downregulated in tumors compared to paired normal liver tissues. Both v1 and v3 transcripts were downregulated in tumors, whereas v2 transcripts were upregulated in tumors compared to normal tissues, suggesting that the latter may be differentially regulated (Figure 3A). Consistent with this idea, in normal livers, v1 and v3 transcripts were positively correlated, whereas v2 did not show a positive correlation with either v1 or v3 transcripts (Figure 3B).

The ratio of v2 and v3 combined to v1 transcripts (v2/v3 versus v1) was measured in all samples. This ratio was highly variable, ranging from 0.02 to 0.25 across the normal samples and from 0.02 to 0.65 in tumors. The median v2/v3 versus v1 ratio was almost two-fold higher in liver tumors (~0.1) than in normal tissues (0.05) (Figure 3Ec), suggesting that tumors might produce relatively more UGT1A_i2 protein.

Examining v2 and v3 abundances separately, we found that median v2 versus v1 and v3 versus v1 ratios were both significantly higher in liver tumors than in normal liver tissues (Figure 3C,D,E(a),E(b)). In normal liver tissues, the median v2 versus v3 ratio was approximately 1 (Figure 3E(d)). Moreover, 42% of livers showed higher v2 than v3, 42% showed higher v3 than v2, and 16% showed similar abundance. These data suggest that these two variant transcripts likely contribute similarly to _i2 protein production in liver tissues(Figure 3C,D). Data were similar for liver tumors, albeit with a slight increase in the median v2 versus v3 ratio (Figure 3E(d)).

Overall, these data reveal that v2 and v3 levels combined are low relative to v1 levels in the liver (approximately 5% of v1 abundance); however, this ratio is elevated in HCC (approximatley 10%). Moreover, v2 and v3 transcripts are expressed at similar levels across the cohort, albeit with considerable interindividual variation.

### 3.3. Expression Profiles of UGT1A Transcripts in Normal Kidney Tissues and Their Deregulation in Kidney Tumor Tissues

**Figure 4:**
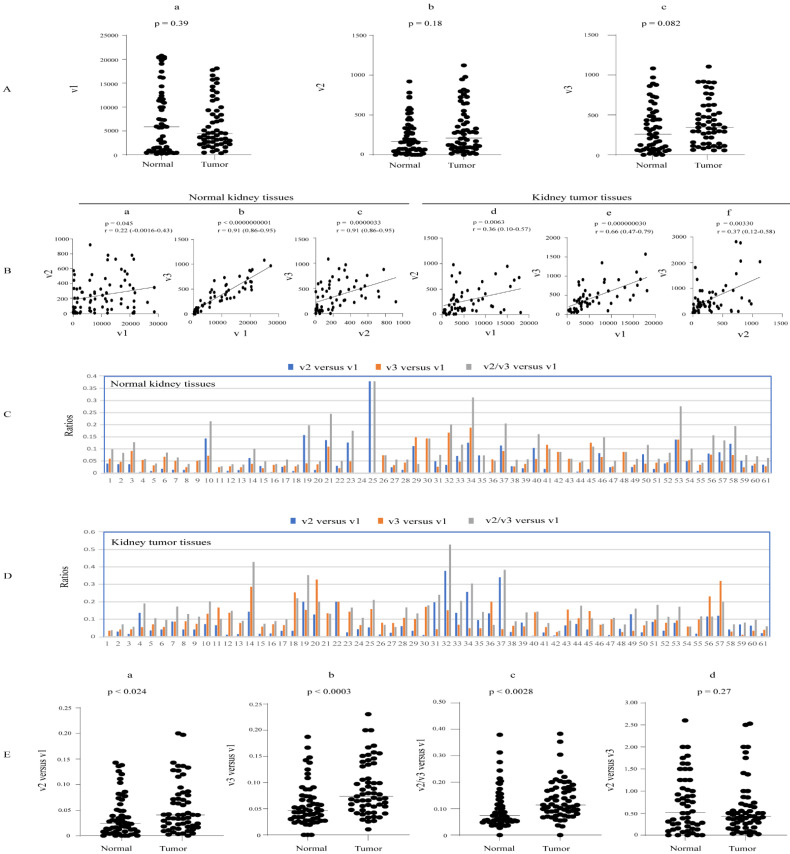
Expression of UGT1A transcripts in kidney tumors and matched adjacent normal kidney tissues. The RNA-seq dataset of 61 paired renal cell carcinoma (RCC) tumor- and adjacent normal kidney tissues (SRP238334) was downloaded from the NCBI database, and the sequence reads of UGT1A transcripts were obtained using the SRA toolkit. Shown are the expression levels of UGT1A_v1, _v2, and _v3 transcripts (**A**) and analyses of their correlation (**B**) and relative abundance (**C**–**E**) in both normal and tumor tissues. Deregulation analysis using Wilcoxon matched-pairs signed rank test (**A**,**E**) and correlation analysis using Spearman ranking test (**B**) were conducted using GraphPad Prism (version 9.1.1). *p* < 0.05 is considered statistically significant. RCC: renal cell carcinoma.

We assessed the abundance of UGT1A transcripts v1, v2, and v3 in an RNA-seq dataset from 61 paired clear cell renal cell carcinoma (RCC) tumors and adjacent normal tissues. All three types of transcripts showed expressions in almost all normal and tumor kidney tissues, with a high expression variability in normal (150-, 60-, and 71-fold, respectively) and tumor (113-, 337-, and 64-fold, respectively) tissues (Table 1, Figure S3). Analysis of the paired samples showed that none of these transcripts were significantly up- or downregulated in kidney tumors compared to matched normal tissues (Figure 4A). All three types of transcripts were positively correlated with each other in both normal and tumor kidney tissues (Figure 4B).

Taking v2 and v3 transcripts together, the median v2/v3 versus v1 expression ratio was significantly higher in kidney tumors at 0.11 (range of 0.03–0.528) than in normal kidney tissues at 0.07 (range of 0.027–0.379) (Figure 4E(c)). Considering v2 and v3 transcript abundances separately, we found that the median v2 versus v1 expression ratio was slightly but significantly higher in tumor tissues (0.04; range of 0.002–0.377) than in normal tissues (0.03; range of 0.002–0.379) (Figure 4C,D,E(a)). The v3 versus v1 expression ratio was two-fold higher in tumors (0.08; range of 0.010–0.327) than in normal tissues (0.04; range of 0.019–0.18) (Figure 4C,D,E(b)). The median v2 versus v3 ratio was approximately 0.5 in both normal and tumor kidney tissues (Figure 4Ed), indicating that v3 transcripts were two-fold more abundant than v2 on average. Moreover, we found that 57% of normal kidneys showed higher v3 than v2 levels, while 33% showed higher v2 than v3, and about 10% showed similar abundance (Figure 4C,D).

Overall, we conclude that v2 and v3 levels are low relative to v1 transcripts (~7%) in normal kidneys, but their relative abundance is elevated in tumors (~11%). Moreover, v3 transcripts are expressed at a higher level than v2 across the cohort, albeit with considerable interindividual variation.

### 3.4. Expression Profiles of UGT1A Transcripts in Normal Colorectal Tissues and Their Deregulation in Colorectal Cancer (CRC)

**Figure 5:**
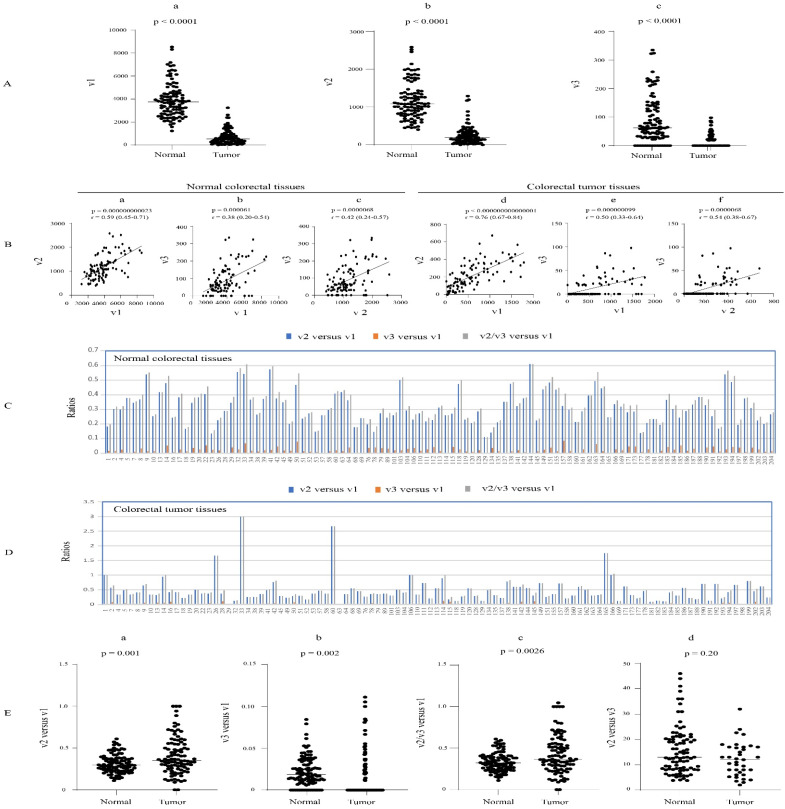
Expression of UGT1A transcripts in CRC tumor- and matched adjacent normal colorectal tissues. The RNA-seq dataset of 103 paired CRC tumor- and adjacent normal colorectal tissues (SRP107326) was downloaded from the NCBI database, and the sequence reads of UGT1A transcripts were obtained using the SRA toolkit. Shown are the expression levels of UGT1A_v1, _v2, and _v3 transcripts (**A**) and analyses of their correlation (**B**) and relative abundance (**C**–**E**) in both normal and tumor tissues. Deregulation analysis using Wilcoxon matched-pairs signed rank test and correlation analysis using Spearman ranking test were conducted using GraphPad Prism (version 9.1.1). *p* < 0.05 is considered statistically significant.

The expression variability and potential dysregulation of UGT1A transcripts v1, v2, and v3 in CRC were assessed using an RNA-seq dataset from 103 paired CRC tumor- and adjacent normal colorectal tissues. The v1 and v2 transcripts were found in almost all normal and tumor colorectal tissues, while v3 transcripts were expressed in 88 normal tissues and 38 CRC tumor tissues. v1 transcripts showed much greater interindividual expression variability in tumors than in normal tissues (150- versus 7-fold). Similarly, v2 abundance was much more variable in tumors than normal tissues (82- versus 6.3-fold) (Table 1, Figure S4). However, v3 variability was not as discrepant between tumors and normal tissues (15- versus 5.3-fold) (Table 1 and Table S4). All three types of transcripts (v1, v2, v3) were highly downregulated (by an average of approximately 10-fold) in CRC tumors compared to matched normal specimens and were positively correlated with each other in both normal and tumor tissues (Figure 5A,B).

Taking v2 and v3 transcripts together, the median v2/v3 versus v1 expression ratio was slightly but significantly higher in CRC tumors (0.37; range of 0.09–2.66) compared to normal colorectal tissues (0.32; range of 0.11–0.61) (Figure 5E(c)). Examining v2 and v3 transcripts separately revealed a very low relative abundance of v3 transcripts. The median v2 versus v3 ratio was approximately 12 in both normal and tumor tissues (Figure 5E(d)). Moreover, 100% of the samples that expressed both variant transcripts showed much higher levels of v2. These data suggest that v2 is the major contributor to the production of UGT1A_i2 proteins in colorectal tissues (i.e., colon, rectum). The v2 versus v1 expression ratio was highly variable in both normal (0.11–0.61) and tumor (0.09–3.00) tissues, with the median ratio significantly increased from 0.29 in normal colorectal tissues to 0.36 in CRC tumors (Figure 5C,D,E(a)). Similarly, the v3 versus v1 expression ratio was highly variable in both normal (0.005–0.08) and tumor (0.018–0.111) tissues, with the median ratio significantly increased from 0.018 in normal colorectal tissues to 0.037 in CRC tumors (Figure 5C,D,E(b)). Overall, we found that v2 transcripts are moderately abundant in both normal and cancerous colorectal tissues, with an average level of about one-third of that of v1 (approximately 36% in tumor tissues and 29% in normal); moreover, several tumor samples showed levels of v2 that were equal to or greater than v1. This relative v2 abundance is much higher than those seen in the liver and kidneys. In contrast, v3 transcripts showed minimal or no expression in colorectal tissues (Figure 5C,D).

We considered that the relatively high v2 versus v1 transcript expression ratio in colorectal tissues may lead to higher UGT1A_i2 versus_i1 protein ratio. This could impact on the UGT1A-medidated glucuronidation pathway in these tissues and potentially influence CRC tumor progression and/or patient survival. We examined this possibility by measuring the variant expression levels, as well as the variant/canonical transcript ratios, across four different CRC tumor stages. However, the levels were not statistically different between stages (Figure S5). The relationship between variant expression or variant/canonical transcript expression ratios and patient survival could not be assessed, as survival data were not available.

### 3.5. Expression Profiles of UGT1A Transcripts in Normal Stomach Tissues and Their Deregulation in Stomach Cancer

Our initial analysis of the normal stomach tissues from the HPA dataset suggested that UGT1A_v2 transcripts were particularly abundant in this tissue (Figure 2). To further characterize this pattern, we quantified all three UGT1A transcripts, v1, v2, and v3, in an RNA-seq dataset of 80 paired stomach cancer and adjacent normal stomach tissues. The v1 and v2 transcripts showed expression in almost all normal and tumor stomach tissues, while v3 transcripts were expressed in 64 normal tissues and 58 tumor tissues (Table 1, Figure S6). v1 transcripts showed very high interindividual expression variation in normal tissues (369-fold) but were less variable in tumors (62-fold). v2 transcripts showed similar variability in normal and tumor tissues (62- and 64-fold, respectively), while v3 transcripts showed an overall lower variability in normal and tumor tissues (21- and 14-fold, respectively) (Table 1, Figure S5).

**Figure 6:**
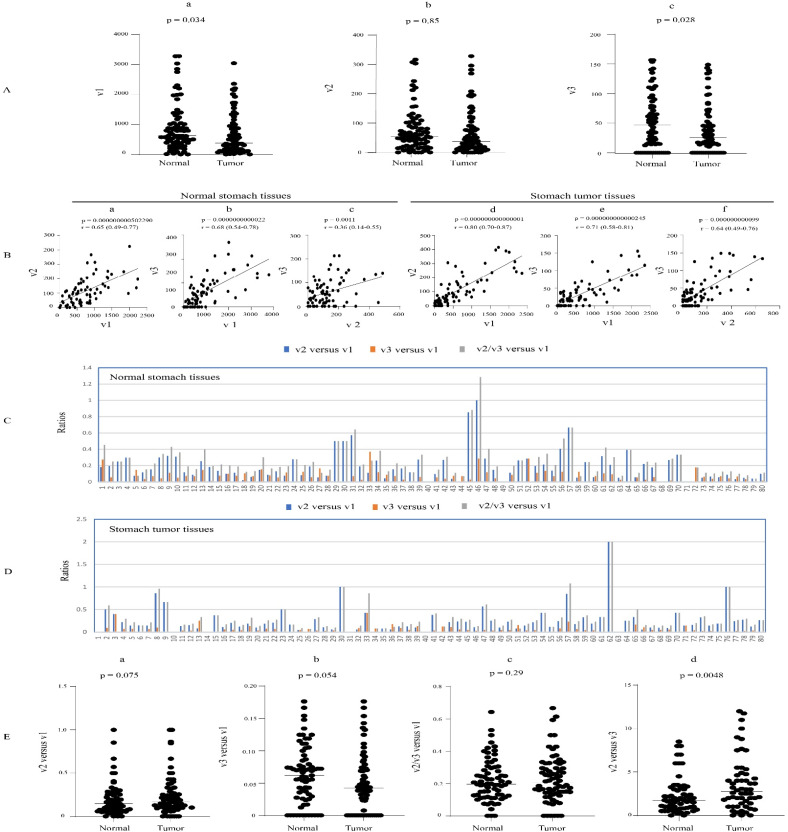
Expression of UGT1A transcripts in stomach tumors and matched adjacent normal stomach tissues. The RNA-seq dataset of 80 paired stomach tumor- and adjacent normal stomach tissues (SRP172499) was downloaded from the NCBI database, and the sequence reads of UGT1A transcripts were obtained using the SRA toolkit. Shown are the expression levels of UGT1A_v1, _v2, and _v3 transcripts (**A**) and analyses of their correlation (**B**) and relative abundance (**C**–**E**) in both normal and tumor tissues. Deregulation analysis using Wilcoxon matched-pairs signed rank test and correlation analysis using Spearman ranking test were conducted using GraphPad Prism (version 9.1.1). *p* < 0.05 is considered statistically significant.

The analysis of the paired samples showed that v1 and v3 transcripts were downregulated in stomach cancer compared to matched normal stomach tissues (Figure 6A(a),A(c)). v2 transcripts also showed a trend of reduced expression in tumors; however, the difference was not significant (Figure 6A(b)). All three types of transcripts were positively correlated with each other in both normal and tumor tissues (Figure 6B). The median v2/v3 versus v1 expression ratio was similar in normal (0.19) and tumor (0.21) stomach tissues (Figure 6E(c)). The median v2 versus v1 expression ratio was similar in normal (0.16) and tumor (0.14) tissues (Figure 6C,D,E(a)), while the median v3 versus v1 expression ratio was also similar in normal (0.06) and tumor (0.04) tissues, respectively (Figure 6C,D,E(b)). Finally, the median v2 versus v3 expression ratio was 1.75 in normal stomach tissues, and this was significantly increased to 2.90 in tumor tissues (Figure 6E(d)). Consistent with the higher median v2 transcript abundance, just over 70% of stomach samples showed a greater v2 than v3 expression (Figure 6C,D).

Taking these data together, we conclude that v2 and v3 transcripts have low-to-moderate abundance in normal and tumor stomach tissues at ~20% of v1 levels; moreover, v2 transcripts predominate over v3 transcripts by around 2–3-fold.

### 3.6. Expression Profiles of UGT1A Transcripts in Normal Esophagus Tissues and Their Deregulation in Esophagus Cancer

**Figure 7:**
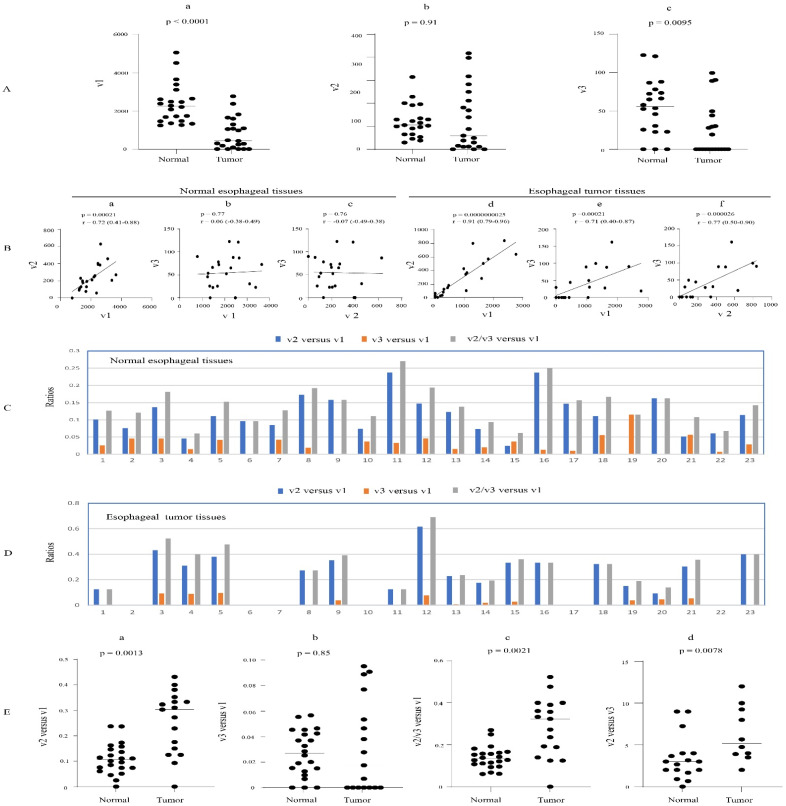
Expression of UGT1A transcripts in esophagus tumors and matched adjacent normal esophagus tissues. The RNA-seq dataset of 23 paired esophagus tumor- and adjacent normal esophagus tissues (SRP193095) was downloaded from the NCBI database, and the sequence reads of UGT1A transcripts were obtained using the SRA toolkit. Shown are the expression levels of UGT1A_v1, _v2, and _v3 transcripts (**A**) and analyses of their correlation (**B**) and relative abundance (**C**–**E**) in both normal and tumor tissues. Deregulation analysis using Wilcoxon matched-pairs signed rank test and correlation analysis using Spearman ranking test were conducted using GraphPad Prism (version 9.1.1). *p* < 0.05 is considered statistically significant.

An RNA-seq dataset from 23 paired esophageal cancer- and adjacent normal tissues was examined to define the expression patterns of UGT1A transcripts v1, v2, and v3. v1 and v2 transcripts were expressed in almost all normal and tumor tissues, while v3 transcripts were expressed in 20 normal tissues and 12 tumor tissues. Both v1 and v2 transcripts showed a greater expression variability in tumors (101- and 60-fold, respectively) than in normal tissues (6.5- and 18-fold, respectively) (Table 1, Figure S7). The v3 transcripts showed a similar degree of variability in normal and tumor tissues (13- and 18-fold, respectively) (Table 1 and Table S6). Comparing matched tumor and normal tissues revealed that v1 and v3 transcripts were significantly downregulated in the tumor tissues (Figure 7A). All three transcript types were positively correlated with each other in tumors, while in normal tissues, only v1 and v2 transcripts showed a positive correlation (Figure 7B).

Taking v2 and v3 transcripts together, the median v2/v3 versus v1 expression ratio was significantly higher in tumors (0.32) than in normal tissues (0.13) (Figure 7E(c)). Examining v2 and v3 separately, we found that v2 transcripts were more abundant than v3 in both normal and tumor tissues, with a median v2 versus v3 expression ratio of 3.1 in normal tissues and 5.2 in tumors (Figure 7E(d)). Consistent with these data, 87% of normal esophagus tissues showed higher v2 than v3 levels (Figure 7C,D). The median v2 versus v1 expression ratio was significantly higher in tumors (0.30) than in normal tissues (0.11) (Figure 7C,D,E(a)). However, the v3 versus v1 expression ratio was not significantly different between tumors and normal tissues (Figure 7C,D,E(b)).

In summary, we find that v2 and v3 transcripts combined have low abundance in normal esophageal tissues at ~13% of v1 levels; however, this is elevated to ~32% in tumors. Moreover, v2 transcripts predominate over v3 transcripts by around 3- to 5-fold (Figure 7E(c),E(d)).

### 3.7. Expression Profiles of UGT1A Transcripts in Normal Bladder Tissues and Their Deregulation in Bladder Cancer

**Figure 8:**
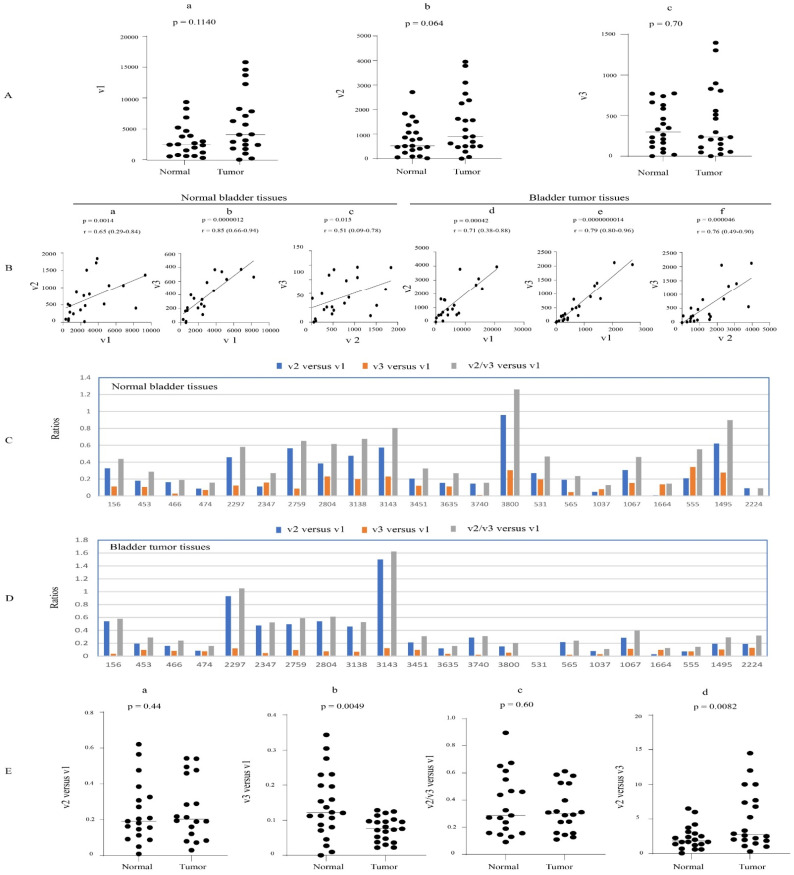
Expression of UGT1A transcripts in bladder tumors and matched adjacent normal bladder tissues. The RNA-seq dataset of 23 paired bladder tumor- and adjacent normal bladder tissues (SRP212702) was downloaded from the NCBI database, and the sequence reads of UGT1A transcripts were obtained using the SRA toolkit. Shown are the expression levels of UGT1A_v1, _v2, and _v3 transcripts (**A**) and analyses of their correlation (**B**) and relative abundance (**C**–**E**) in both normal and tumor tissues. Deregulation analysis using Wilcoxon matched-pairs signed rank test and correlation analysis using Spearman ranking test were conducted using GraphPad Prism (version 9.1.1). *p* < 0.05 is considered statistically significant.

The expression profiles of UGT1A transcripts v1, v2, and v3 were assessed using an RNA-seq dataset from 22 paired bladder cancer- and adjacent normal bladder tissues. v1, v2, and v3 transcripts were expressed in almost all normal and tumor bladder tissues, with a high expression variability in normal (108-, 131-, 133-fold, respectively) and tumor (142-, 94-, and 88-fold, respectively) tissues (Table 1, Figure S8). v1, v2, and v3 transcripts were positively correlated with each other in both normal and tumor tissues; none of the transcript types were significantly differentially expressed in tumor vs. normal tissues (Figure 8A,B).

Considering transcripts v2 and v3 together, the median v2/v3 versus v1 expression ratio was similar in normal and tumor tissues at 0.28 and 0.3, respectively (Figure 8E(c)). Examining the variant transcripts separately, we showed that v2 levels were higher than v3 levels in both normal and tumor tissues, with the median v2 versus v3 expression ratio significantly increased from 2.0 in normal tissues to 2.8 in tumors (Figure 8E(d)). Consistent with this, 82% of normal tissues showed a higher v2 than v3 expression (Figure 8E(d)). The v2 versus v1 expression ratio was highly variable in both normal (0.008–0.95) and tumor (0.029–1.50) tissues, with a similar median ratio in normal (0.206) and tumor (0.212) tissues (Figure 8C,D,E(a)). The v3 versus v1 expression ratio was also highly variable in both normal (0.09–1.26) and tumor (0.109–1.62) tissues, with the median ratio significantly decreased from 0.121 in normal tissues to 0.075 in tumor tissues (Figure 8C,D,E(b)).

In summary, v2 and v3 transcripts combined have moderate abundance in normal and tumor tissues at up to 30% of v1 levels; moreover, v2 transcripts predominate over v3 transcripts by around 2- to 3-fold.

### 3.8. Discovery of Novel UGT1A Transcripts with 3′ Variant Splicing 

**Figure 9:**
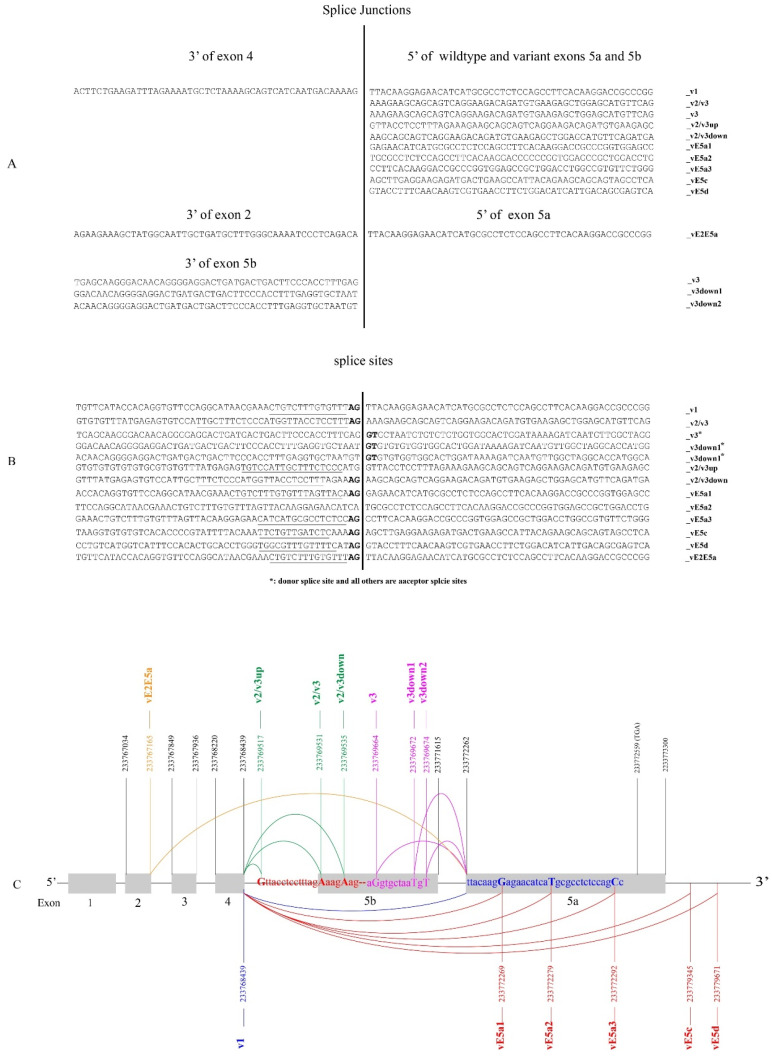
Discovery of novel UGT1A transcripts with novel 3′ ends. The UGT-captureSeq data (SRP073607) were downloaded from the NCBI database and analyzed with a focus on identifying novel UGT1A transcripts with 3′ differences, as described in Materials and Methods. Shown are the splice junctions (**A**) and splice sites (**B**) for ten novel UGT1A transcripts (as indicated) that were identified from UGT-CaptureSeq samples. (**C**) A diagram showing the exon structure of the *UGT1A* locus and both wildtype and variant splicing events that generate three known UGT1A transcripts (v1, v2, v3) and ten novel UGT1A transcripts with 3′ differences (as indicated). The genomic positions of wildtype and variant exons are annotated according to the human genome GRCH/hg38 assembly. Also indicated include variant splicing 1) ligating exon 4 to novel downstream exons (RED), 2) ligating exon 5a to novel upstream exons (PURPLE), 3) between exon 4 and variant exon 5b (GREEN) are also indicated.

As depicted in Figure 1B, the splicing of exon 4 to exon 5a and 5b generates UGT1A_v1 and _v2/v3 transcripts, respectively (Figure 1B). Any further UGT1A transcripts that might affect the 5a/5b-exon region remains unknown. To investigate this, our bioinformatic analysis of the fifteen UGT-CaptureSeq datasets identified over 5000 sequence reads that had a sequence attached at the 3′ end of exon 4 or at the 5′ end of exon 5a or 5b (Tables S8–S10). As expected, most of these sequence reads were derived from transcripts v1, v2, and v3; however, we found seven novel splice junctions in which exon 4 was spliced to a downstream novel sequence using cryptic acceptor sites (Figure 9A,B). Briefly, exon 4 was spliced to a novel sequence within vE5a1, vE5a2, or vE5a3 or downstream (vE5c, vE5d) of exon 5a, generating novel UGT1A transcripts that presumably contain exons 1–4 and a novel exon 5 (Figure 9C). Exons vE5a1, vE5a2, and vE5a3 started at 8, 18, and 31 nucleotides downstream of the 5′ end of exon 5, and thus, they lacked the 5′ 8, 18, and 31 nucleotides of exon 5a, respectively (Figure 9C). Exons vE5c and vE5d started at 7054 and 7405 nucleotides downstream of the 3′ end of exon 5a, and thus, they contained novel sequences that were completely different from exon 5a (Figure 9C). Finally, exon 4 was spliced to 14 nucleotides upstream (v2/v3up) or 4 nucleotides downstream (v2/v3down) of the 5′ end of exon 5b, generating novel UGT1A transcripts that had an extended or shortened exon 5b or 5bv (Figure 9C).

The splicing of exon 5bv between exons 4 and 5a generates UGT1A_v3 transcripts (Figure 1B). In the present study, exon 5a was found to be spliced to 8 (v3down1) or 10 (v3down2) nucleotides downstream of the 3′ end of exon 5bv, generating novel UGT1A transcripts with an extended exon 5bv (Figure 9A,C). Finally, exon 5a was also found to be spliced to the 3′ end of exon 2 (vE2E5a), generating novel UGT1A transcripts that had only three canonical exons (i.e., E1, E2, E5a) (Figure 9A,C).

Overall, we found a series of novel splice junctions that predict ten novel different types of UGT1A transcripts with 3′ abnormalities. Using the SRA platform and transcript-specific probes, we quantified these ten novel transcripts in the UGT-enriched CaptureSeq samples. All ten transcripts were found in UGT-CaptureSeq samples, with three (v3down1, v2/v3down, vE5a1) being found in more than half samples (Table S11). v2/v3down was the most common variant that was found in all fifteen UGT-CaptureSeq samples (Table S11). All sequence reads for the ten novel transcripts that were identified from the fifteen UGT-Capture-Seq samples were presented in Figures S9 and S10. To further verify the expression of these novel variants, we assessed whether they were also present in RNA-seq samples of the six cancer types that were analyzed in this study, including 354 paired normal and tumor tissues (Table S12). Eight (v3down1, v3down2, v2/v3up, v2/v3down, vE5a1, vE5c, vE5d, vE2E5a) of the ten novel transcripts were found in these RNA-Seq samples (Tables S2–S7 and S12). Again, transcript v2/v3down was the most common variant and was found in 21% (76/354) of normal tissues and 13% (44/354) of tumor tissues. This transcript was common in normal liver (33%), kidney (25%), and colorectal (30%) tissues, as well as in HCC tumors (30%), but relatively less so in RCC (13%) and CRC (3%) tumors (Table S12). Overall, the identification of eight of the ten novel variant junctions in many samples in different RNA-seq datasets, derived using different methodologies and platforms, supports that the transcripts are likely expressed in normal and tumor tissues.

Eight of the ten novel transcripts are predicted to encode variant UGT1A proteins that have a similar structure to UGT1A_i2 proteins (Table S13). Whether these novel proteins have a similar role to i2 proteins that inhibit the activity of i1 proteins remains to be investigated. For example, the predicted v2/v3down proteins have the N-terminal part of UGT1A_i1 proteins that are encoded by exons 1–4 and a novel 39-aa C-terminal peptide that is encoded by exon v2/v3down (Table S13). We used the PepQuery2 platform to interrogate proteomes for spectra matching the novel 39aa C-terminal peptide. Matches assessed as confident (passing all PepQuery filtering steps) were found in 6 of 18 assessed proteomic datasets (GTEx_32_Tissues Proteome, Gastric Cancer Proteome, Gastric Cancer Glycoproteome, UCEC Discovery Study proteome, Prospective Ovarian JHU Proteome, and Academica_Sinica_LUAD100 Proteome) (http://www.pepquery.org, accessed on 1 November 2023) [39]. This finding provides support for the translation of this variant transcript. However, further confirmatory studies will be required to validate these variant transcripts, including RT-PCR and Sanger sequencing.

## 4. Discussion

The *UGT1A* locus shows complex canonical and alternative splicing that remains incompletely understood [15,16,18,19]. The v2 and v3 transcripts are the only UGT1A transcripts that are known to be translated into proteins (i.e., UGT1A_i2) [17,18,19,20,21,22,23,24,25,26]. UGT1A_i2 proteins have no transferase activity, but they can inhibit UGT1A_i1 proteins via oligomerization and interact with other metabolic enzymes [21,22,23,24,25,26,40]. Hence, the level of v2 and v3 variants relative to canonical v1 transcripts may be an important determinant of glucuronidation activity and also influence other aspects of cellular metabolism [24]. This study aimed to address gaps in our understanding of UGT1A transcripts, including interindividual expression variability, differences between v2 and v3 variant expression, and their potential dysregulation in cancer.

The initial interrogation of the 27-tissue HPA panel [31] revealed the expression of v1, v2, and v3 transcripts in liver, kidney, and gastrointestinal tissues (esophagus, stomach, duodenum, small intestine, colon) and several other tissues, which is broadly consistent with the findings of a previous study of 14 human tissues using non-quantitative RT-PCR [18]. As expected, v2 and v3 levels were significantly lower than v1 levels in almost all tissues, with the exception of testis, which showed preferential expression of the v2 variant. Given that v2 and v3 transcripts encode the same proteins, most previous studies reported only their combined abundance [18,19,27,28,29]. Therefore, the relative expression levels of v2 and v3 and their potential differential expression remain unknown. In the present study, we quantified v2 and v3 separately, revealing higher v2 than v3 levels in the majority of HPA tissues. However, as discussed below, the subsequent analysis of RNA-seq datasets with larger tissue cohorts indicated that v2 predominance was tissue-selective and occurred mainly in gastrointestinal tissues. 

Guided by the initial HPA tissue profiling, we measured the abundance of UGT1A transcripts v1, v2, and v3 using RNA-seq datasets generated from large cohorts of paired normal and tumor samples from the liver, kidney, colon/rectum, stomach, esophagus, and bladder. Of these six tissues, liver and kidney tissues showed the lowest levels of v2 and v3 transcripts relative to v1. In normal and cancerous liver and kidney samples, median v2/v3 versus v1 ratios ranged from 5 to 11%, suggesting that transcripts encoding _i2 proteins may be kept relatively low in these important detoxifying tissues. 

Liver tumors showed significant downregulation of v1 and v3, but not v2 transcripts, relative to the paired normal tissues. Moreover, the v2 versus v1 ratio was increased several folds in tumors. This pattern of dysregulation (reduced v1 and elevated v2 levels) might be expected to lead to a significant reduction in the glucuronidation capacity in HCC. In contrast to liver, kidney tumors (RCC) did not show downregulation of v1, v2, or v3 transcripts relative to normal samples; however, the v2/v3 versus v1 ratio was significantly increased in RCC. 

UGT genes show a high interindividual expression variability, particularly in the liver, because their levels are dynamically regulated by inducible transcription factors that respond to hormones, dietary chemicals, drugs, and other molecules [2]. Our analysis of large sample sets gave further insight into the interindividual variability of UGT1A transcript expression. Notably, all three UGT1A transcript types showed much greater variability in HCC than in normal liver tissues. For example, the variation in v1 expression increased dramatically from 17-fold in normal livers to 1697-fold in HCC, suggesting that tumors are particularly susceptible to UGT1A dysregulation. The regulatory mechanisms of the expression changes between normal and tumor samples are unknown. They may simply reflect the generalized dysregulation of tissue-specific gene expression due to the dedifferentiation of cancer cells [2]. However, the possibility of gene-specific regulatory events cannot be dismissed.

Gastrointestinal tissues showed higher median v2/v3 versus v1 ratios than those in the liver and kidney (i.e., 36%, 19%, and 32% in normal colon, stomach, and esophagus, respectively). This suggests a greater potential for the modulation of glucuronidation activity by the production of UGT1A_i2 proteins in the gut. All three UGT1A transcript types were robustly downregulated in CRC relative to normal colorectal tissues. Stomach and esophageal cancers showed significant downregulation of v1 and v3 transcripts relative to normal tissues, with esophagus also showing an increase in the v2 versus v1 ratio. These changes may be expected to reduce the glucuronidation capacity in tumor cells; however, whether this is a functionally relevant process in tumor pathogenesis is not currently clear. Our preliminary results showed no correlation between the v2 versus v1 ratio and CRC tumor stage; however, our ability to assess such relationships in this study was limited by small cohort sizes. Further studies with larger cohorts could be warranted. 

A general observation from our analysis of six tissue types was that the relative expression of v2 and v3 transcripts (v2 versus v3 ratio) was tissue-specific. In the liver, the median v2 versus v3 ratio was about 1.0, indicating similar abundance. In contrast, in kidneys, the v2 versus v3 ratio was approximately 0.5, indicating that v3 transcripts were about twice as abundant as v2. In all three gastrointestinal tissues examined, v2 levels were several folds higher than v3. Specifically, the median v2 versus v3 ratio was approximately 12 in normal and cancerous colorectal tissues, but relatively low (1.7–5.0) in normal and cancerous stomach and esophageal tissues. These tissue-specific patterns were not evident in the much smaller HPA datasets (generally only 3–5 samples), emphasizing the importance of analyzing large cohorts.

Interestingly, the v2 transcripts also showed a different pattern of dysregulation in tumors compared to v1 and v3 transcripts. For example, both v1 and v3 transcripts were downregulated in liver, colorectal, stomach, and esophageal cancers, whereas v2 transcripts were only downregulated in CRC and were upregulated in liver cancer. The tissue specificity of v2 versus v3 ratios and the observation that v2 transcripts show different patterns of dysregulation relative to v1 and v3 in cancer contexts suggest together that v2 transcripts are differently regulated. The v2 variant has a novel 3′UTR sequence that is associated with exon 5b. In contrast, v3 and v1 transcripts share the exon5a-associated 3′UTR sequence. Multiple miRNAs are known to target the exon 5a-associated 3′UTR and thus regulate the levels of the v1 transcripts and i1 proteins [41]. Thus, it is possible that v2 transcripts are differentially regulated via miRNA-mediated destabilization or other post-transcriptional mechanisms that differentially target the v2- versus v1/v3-specifc 3′UTRs. Overall, the role of post-transcriptional mechanisms in the differential regulation of v1, v2, and v3 transcripts is likely to be complex and requires further study.

Finally, we reported the discovery of ten novel UGT1A transcripts that were generated using cryptic splice sites within or adjacent to exons E5a, E5b, or E5bv. Most of these variants are predicted to encode proteins with a similar structure to _i2 proteins (i.e., intact N-terminal regions and short C-terminal peptides encoded by novel terminal exons). Moreover, tentative evidence for the translation of the most common novel variant (v2/v3down) was obtained from public proteomic datasets [39]. While the novel variants were rarer than v2 and v3 transcripts in all assessed tissues, their identification does further highlight the complexity and diversity of the UGT1A transcriptome and proteome.

Several new questions about UGT1A variant transcripts are prompted by this study. The first is whether v2 levels are differently regulated by miRNAs or RNA-binding proteins compared to the v1 and v3 transcripts. A second, related, question is whether variant transcripts show different translational regulation. Given that miRNAs regulate both mRNA stability and translation efficiency, it is possible that transcripts are differentially sensitive to miRNA-mediated translational inhibition [41]. This could lead to a higher production of the _i2 proteins and thus a greater inhibition of glucuronidation. Both questions could be addressed using methods that we and others have reported previously [2,41]. The third question emerges from a limitation in our study: because we analyzed exon 4/5 splice junctions and used short-read RNA-seq data, we cannot determine which UGT1A isoforms (e.g., UGT1A1-1A10) the reads represent. The variability in v1, v2, and v3 transcript abundance is likely to be a product of multiple processes including differential transcription, splicing, and transcript stability. Unfortunately, the contribution of promoter-specific transcriptional regulation cannot be assessed without quantifying the transcripts at the isoform level. Variant-specific RT-PCR strategies as previously reported could be applied to address this issue in the future [18,27,29].

## 5. Conclusions

This study comprehensively assessed the differential and variable expression of three types of UGT1A transcripts (v1, v2, v3) in human tissues and their deregulation in human cancers. The high v2 and v3 expression variability in different types of tissues suggests that their impacts on glucuronidation is tissue-specific and may be relevant to personalized drug metabolism through the UGT1A conjugation pathway. Moreover, we found higher v2 and v3 abundance relative to v1 transcripts in many types of tumors, which might impact tumor pathogenesis and treatment through altered clearance of growth regulatory endobiotics and anticancer drugs. Finally, the discovery of a set of novel variant UGT1A transcripts with novel 3′ terminal exons further highlights the complexity and diversity of the UGT1A transcriptome and proteome.

## Figures and Tables

**Table 1:** RNA-seq datasets analyzed in this study with the number of tissues that expressed three types of UGT1A transcripts and their individual expression variability in both paired tumor and adjacent normal tissues.

RNA-seq Dataset	No. of Paired Tissues	Normal Tissues						Tumor Tissues						SRA Accession No.
		v1		v2		v3		v1		v2		v3		
		No.	Fold	No.	Fold	No.	Fold	No.	Fold	No.	Fold	No.	Fold	
Liver cancer	65	65	17	64	236	65	14	64	1697	63	454	61	138	SRP401130
Kidney cancer	61	61	150	55	60	58	71	61	113	60	337	61	64	SRP238334
Colorectal cancer	103	103	7.0	103	6.3	88	15	102	150	101	82	38	5.3	SRP107326
Stomach cancer	80	78	369	74	62	64	21	72	62	78	64	58	14	SRP172499
Esophagus cancer	23	23	6.5	22	18	20	13	18	101	20	60	12	18	SRP193095
Bladder cancer	22	22	108	22	131	21	133	21	142	21	94	21	88	SRP212702

## Data Availability

RNA-seq datasets assessed by this study are available from the Sequence Read Archive (SRA) database (https://www.ncbi.nlm.nih.gov/sra) (Accessed on 1 June 2023).

## Supplementary Materials

The following supporting information can be downloaded at https://www.mdpi.com/article/10.3390/cancers16020353/s1: Figure S1: Expression profiles of UGT1A transcripts in normal human tissues; Figure S2: Expression profiles of UGT1A transcripts in HCC (hepatocellular carcinoma) tumor- and matched adjacent normal liver tissues; Figure S3: Expression profiles of UGT1A transcripts in RCC (renal cell carcinoma) tumor- and matched adjacent normal kidney tissues; Figure S4: Expression profiles of UGT1A transcripts in CRC (colorectal carcinoma) tumor- and matched adjacent normal colorectal tissues; Figure S5: Assessment of the difference in the expression levels of variants or the variant/canonical transcript expression ratios across different tumor stages; Figure S6: Expression profiles of UGT1A transcripts in stomach cancer- and matched adjacent normal stomach tissues; Figure S7: Expression profiles of UGT1A transcripts in esophagus cancer- and matched adjacent normal esophagus tissues; Figure S8: Expression profiles of UGT1A transcripts in bladder cancer- and matched adjacent normal bladder tissues; Figure S9: Sequence reads containing specific splice junctions of novel UGT1A transcripts; Figure S10: Sequence reads containing the specific splice junction of novel transcript v2/v3down; Table S1: Sequence reads of wildtype and variant UGT1A transcripts that were found in the HPA RNA-Seq datasets; Table S2: Sequence reads of wildtype and variant UGT1A transcripts that were found in RNAseq datasets of HCC tumor- and matched adjacent normal tissues; Table S3: Sequence reads of wildtype and variant UGT1A transcripts that were found in RNAseq datasets of RCC tumor- and matched adjacent normal tissues; Table S4: Sequence reads of wildtype and variant UGT1A transcripts that were found in RNAseq datasets of CRC tumor- and matched adjacent normal tissues; Table S5: Sequence reads of wildtype and variant UGT1A transcripts that were found in RNAseq datasets of stomach tumor- and matched adjacent normal tissues; Table S6: Sequence reads of wildtype and variant UGT1A transcripts that were found in RNAseq datasets of esophagus tumor- and matched adjacent normal tissues; Table S7: Sequence reads of wildtype and variant UGT1A transcripts that were found in RNAseq datasets of bladder tumor- and matched adjacent normal tissues; Table S8: Analysis of UGT-CaptureSeq data (GSE80463) identified sequence reads that show upstream sequences were spliced to exon 4; Table S9: Analysis of UGT-CaptureSeq data (GSE80463) on identified sequence reads that show that upstream sequences were spliced to exon 5a; Table S10: Analysis of UGT-CaptureSeq data (GSE80463) on identified sequence reads that show that upstream sequences were spliced to exon 5a; Table S11: The number of sequence reads that were identified for wildtype and variant UGT1A transcripts in UGT-CaptureSeq samples (SRP073607); Table S12: The numbers of tumor- and matched normal tissues expressing wildtype and variant UGT1A transcripts that were found in six different types of human cancers; Table S13: UGT1A transcripts and proteins.
