# Supplementary material for: A Comprehensive Bioinformatic Analysis of RNA-seq Datasets Reveals a Differential and Variable Expression of Wildtype and Variant UGT1A Transcripts in Human Tissues and Their Deregulation in Cancers
Source: Cancers (Basel). 2024 Jan 13;16(2):353. doi: 10.3390/cancers16020353 (PMC10814044; doi:10.3390/cancers16020353)
Supplement: Supplementary file 1 [file cancers-16-00353-s001.zip › Supplemental Figures and Tables.pdf]

A Comprehensive Bioinformatic Analysis of RNA-seq Datasets Reveals Differential and Variable Expression of Wildtype and Variant UGT1A Transcripts in Human Tissues and Their Deregulation in Cancers

Dong Gui Hu*, Shashikanth Marri, Julie-Ann Hulin, Ross A McKinnon, Peter I Mackenzie, Robyn Meech

A

B

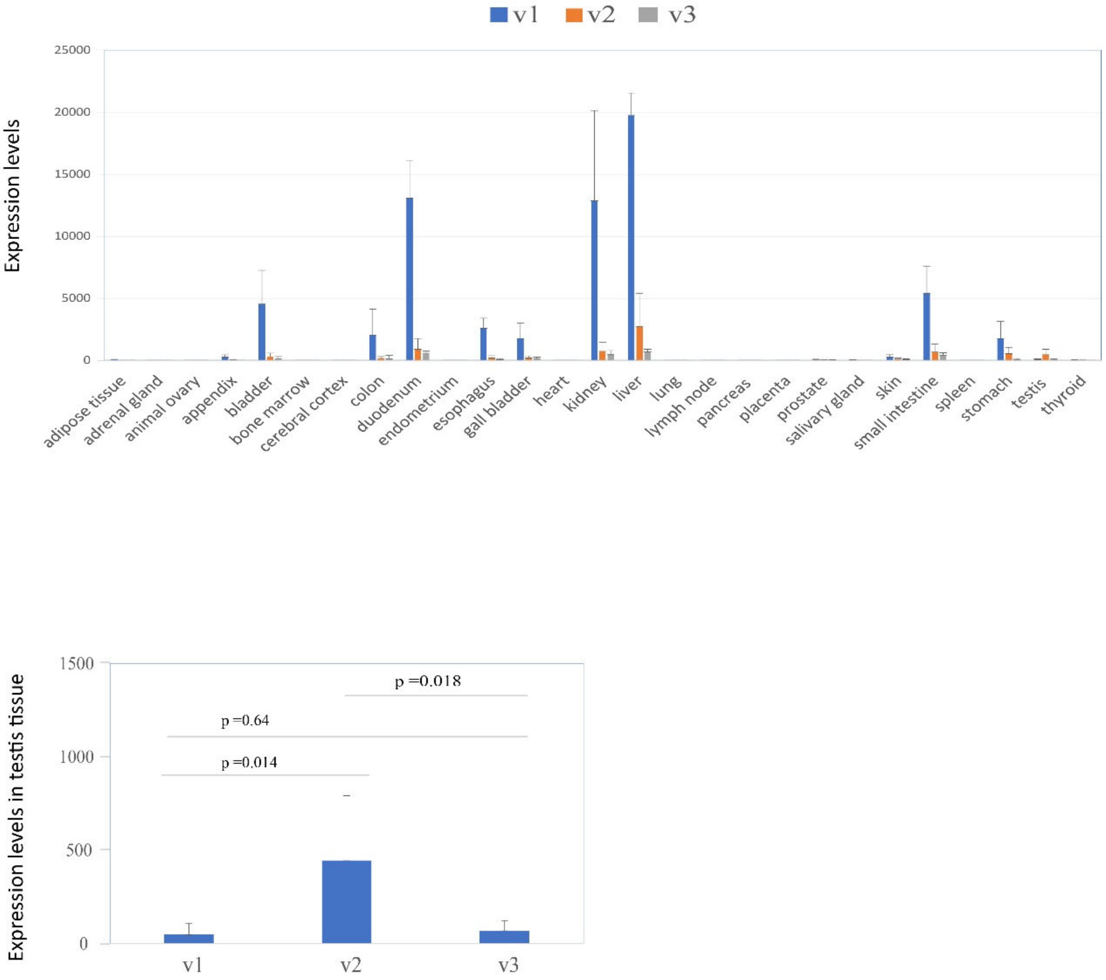

Figure S1. Expression profiles of UGT1A transcripts in normal human tissues. The RNA-seq dataset of the Human Protein Atlas (HPA) was downloaded the NCBI database and the sequence reads of UGT1A transcripts were obtained using the SRA toolkit. Shown are the expression levels of UGT1A_v1, _v2, and _v3 transcripts in 27 different human tissues as indicated (A) and the expression levels of these transcripts in testis were highlighted (B).

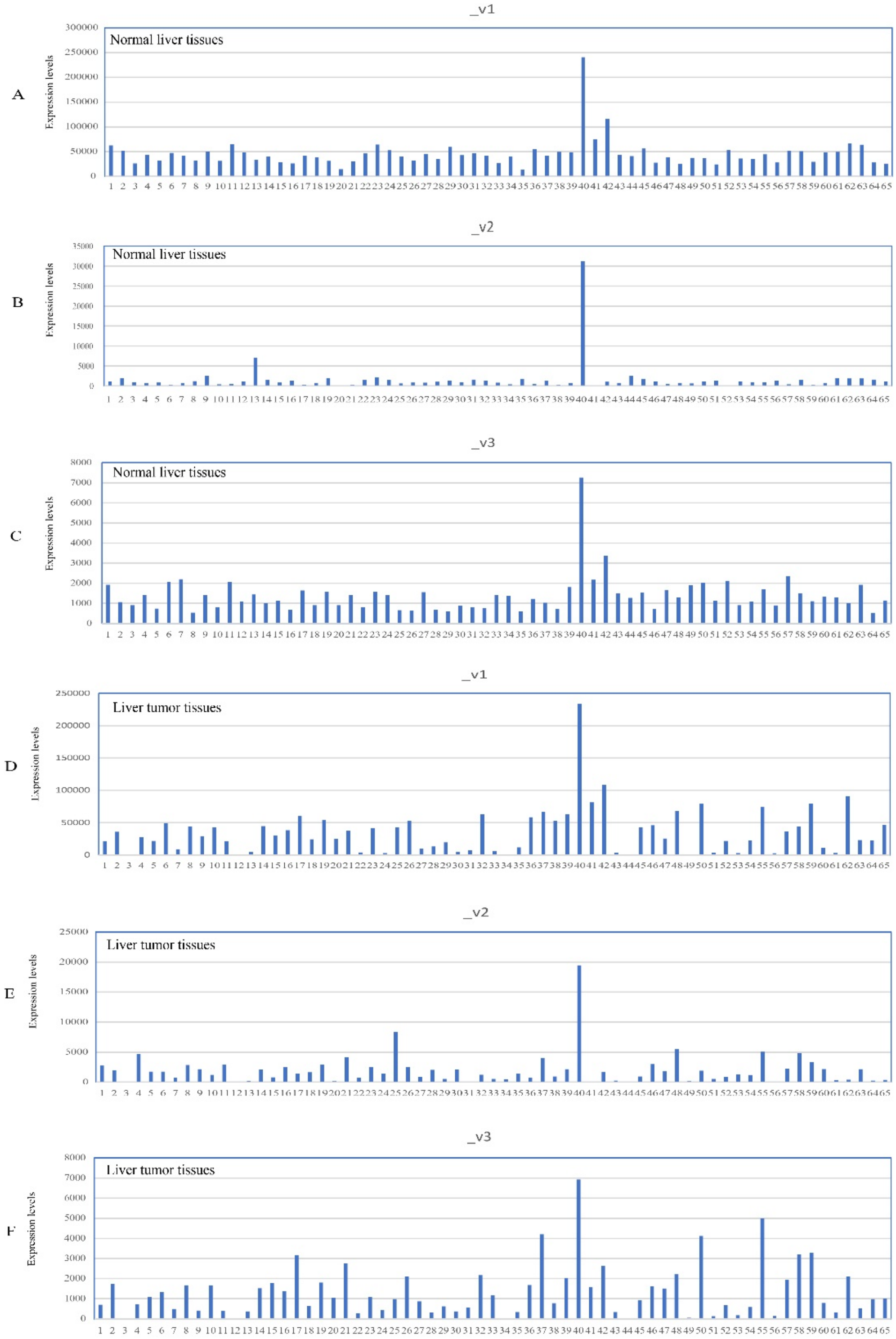

Figure S2. Expression profiles of UGT1A transcripts in HCC (hepatocellular carcinoma) tumor and matched adjacent normal liver tissues. The RNA-seq dataset of 65 paired HCC tumor and adjacent normal liver tissues was downloaded the NCBI database and the sequence reads of UGT1A transcripts (_v1, _v2, _v3) were obtained using the SRA toolkit. Shown are the expression levels of UGT1A_v1, _v2, and _v3 transcripts in normal liver tissues (A-C) and HCC tumors (D-F).

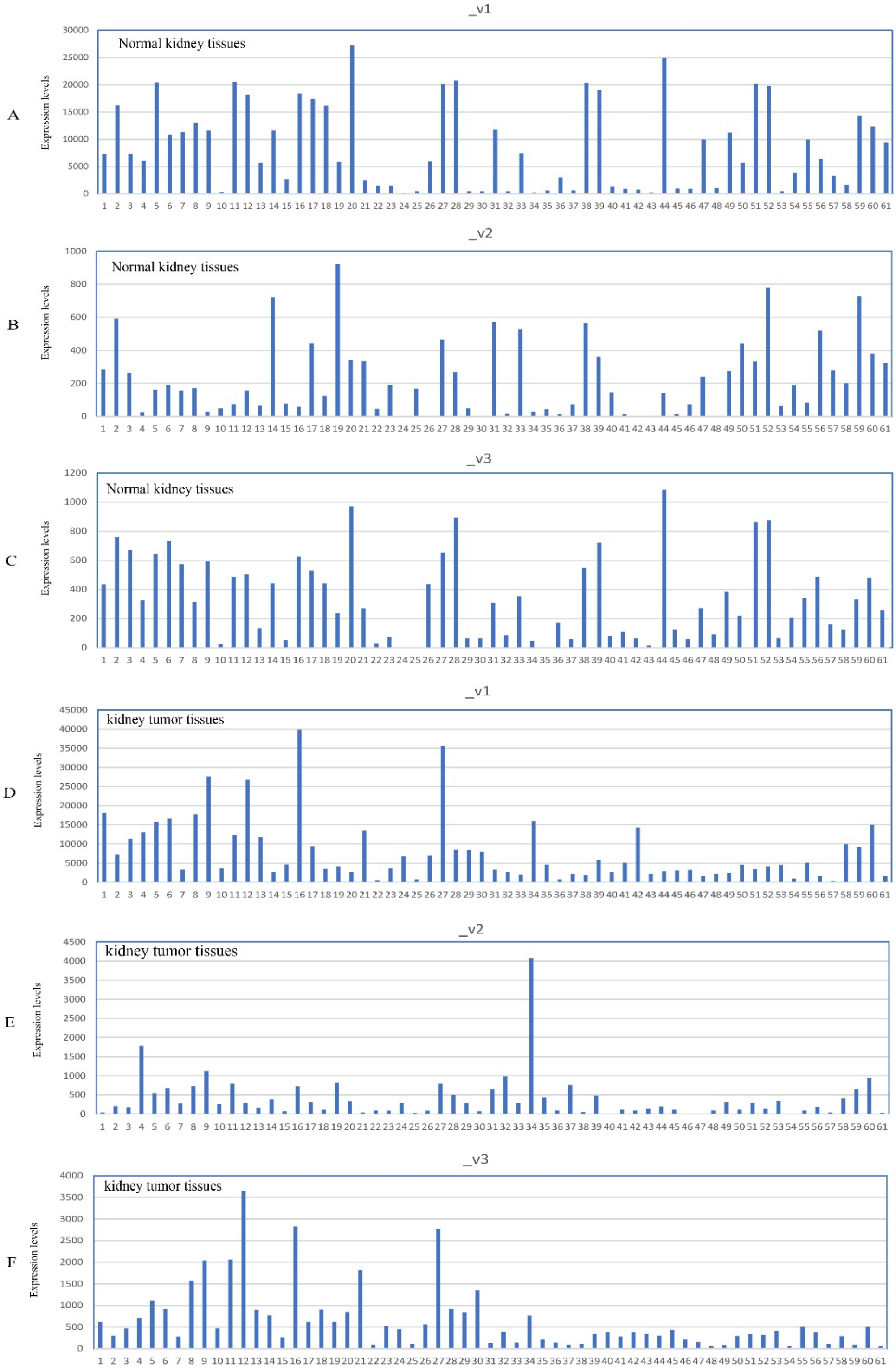

Figure S3. Expression profiles of UGT1A transcripts in RCC (renal cell carcinoma) tumor and matched adjacent normal kidney tissues. The RNA-seq dataset of 61 paired RCC tumor and adjacent normal kidney tissues was downloaded the NCBI database and the sequence reads of UGT1A transcripts (_v1, _v2, _v3) were obtained using the SRA toolkit. Shown are the expression levels of UGT1A_v1, _v2, and _v3 transcripts in normal liver tissues (A-C) and RCC tumors (D-F).

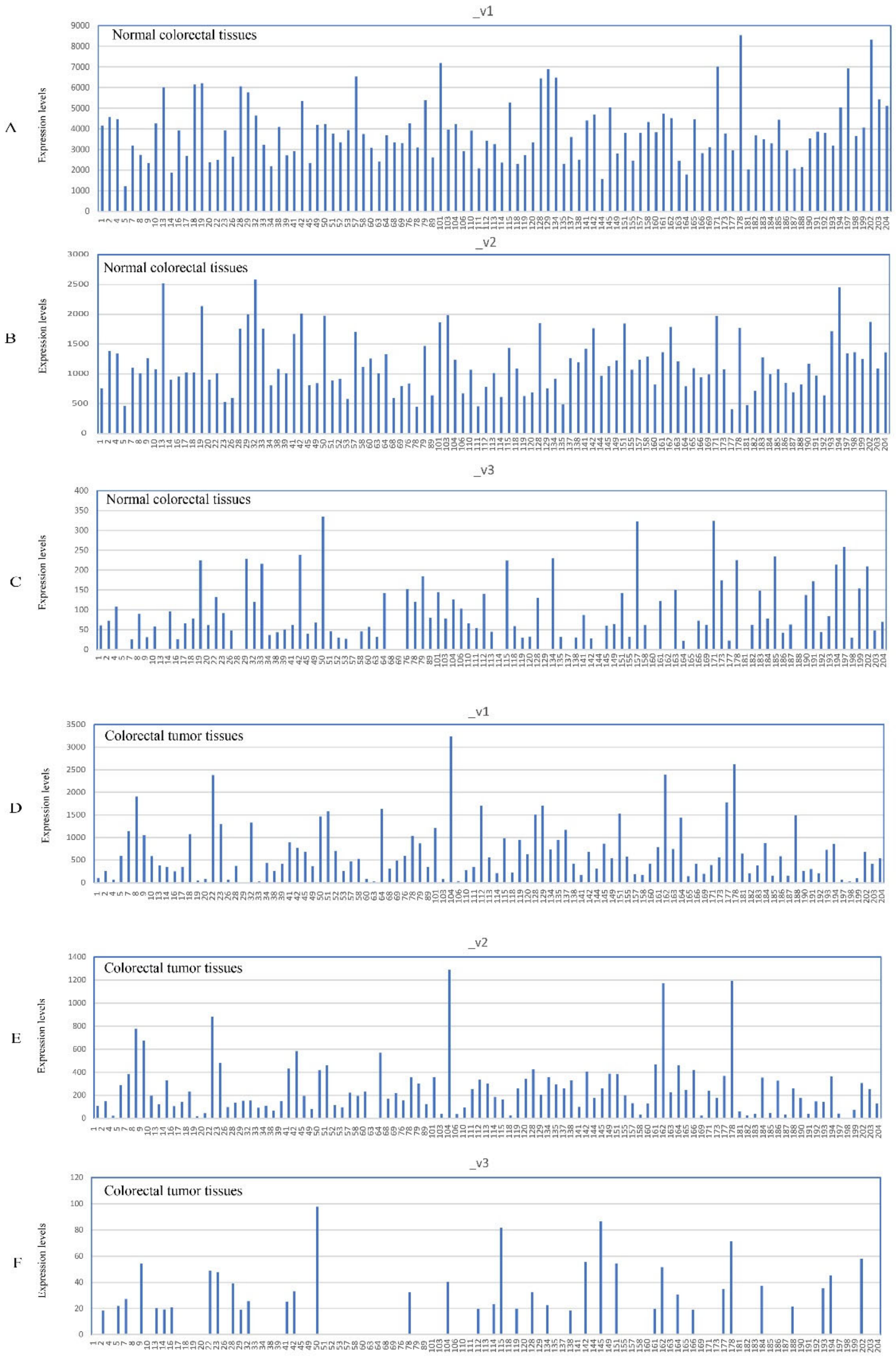

Figure S4. Expression profiles of UGT1A transcripts in CRC (colorectal carcinoma) tumor and matched adjacent normal colorectal tissues. The RNA-seq dataset of 103 paired CRC tumor and adjacent normal colorectal tissues was downloaded the NCBI database and the sequence reads of UGT1A transcripts (_v1, _v2, _v3) were obtained using the SRA toolkit. Shown are the expression levels of UGT1A_v1, _v2, and _v3 transcripts in normal liver tissues (A-C) and RCC tumors (D-F).

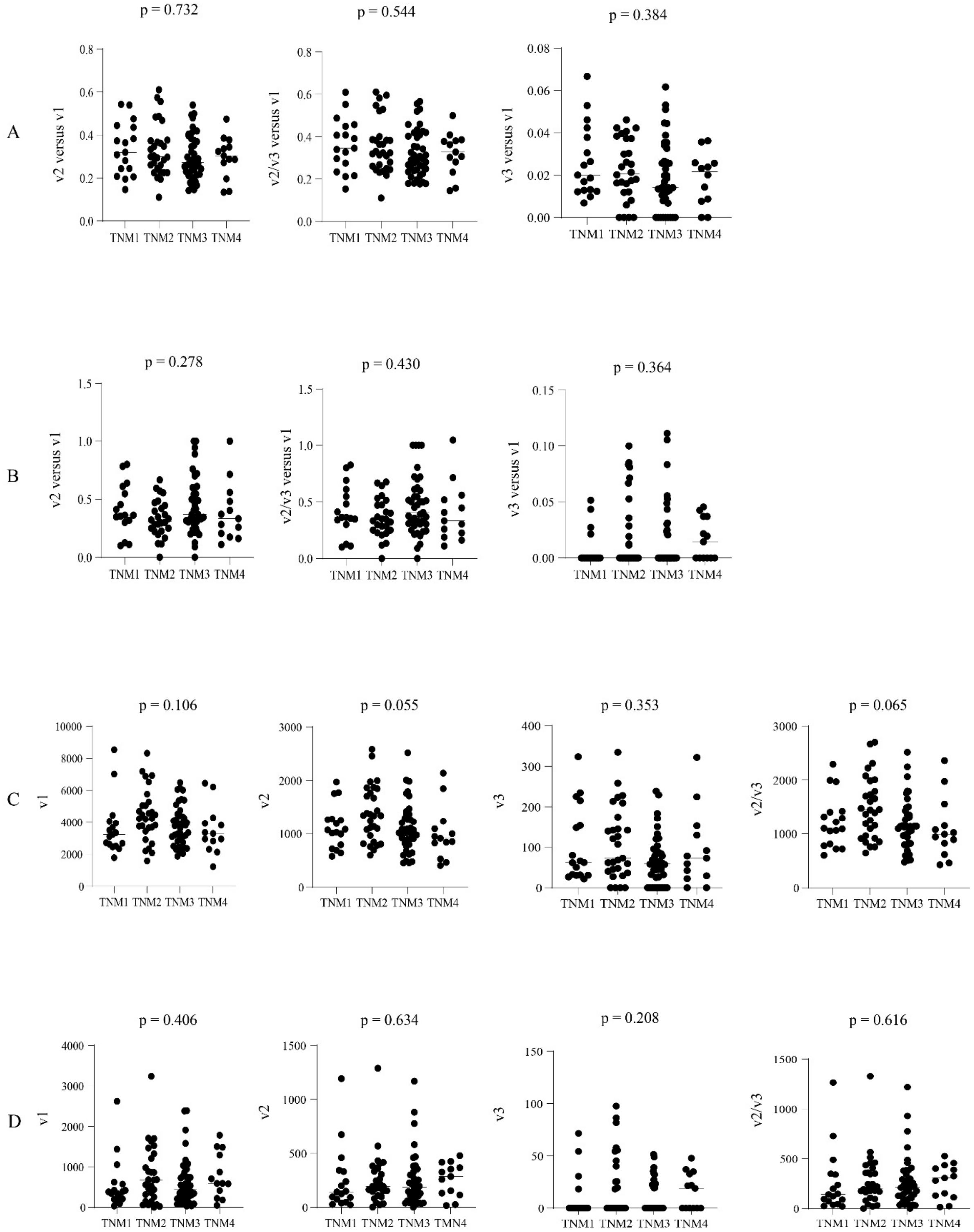

Figure S5. Assessment of the difference in the expression levels of variants or the variant/canonical transcript expression ratios across different tumor stages. The expression levels of UGT1A transcripts (v1, v2, v3) of 103 paired colorectal cancer and adjacent normal tissues and the tumor stages of all 103 colorectal patients were obtained from the SRA database. The potential difference in 1) the expression ratios (v2 versus v1, v2/v3 versus v1, v3 versus v1) across tumor stages (TNM1-4) in normal (A) and tumor (B) tissues, and 2) the expression levels of variants (v1, v2, v2/v3, v3) across tumor stages in normal (C) and tumor (D) tissues were assessed using ANOVA analysis (Kruskal_Wallies Test). A p value of < 0.05 is considered as statistically significant.

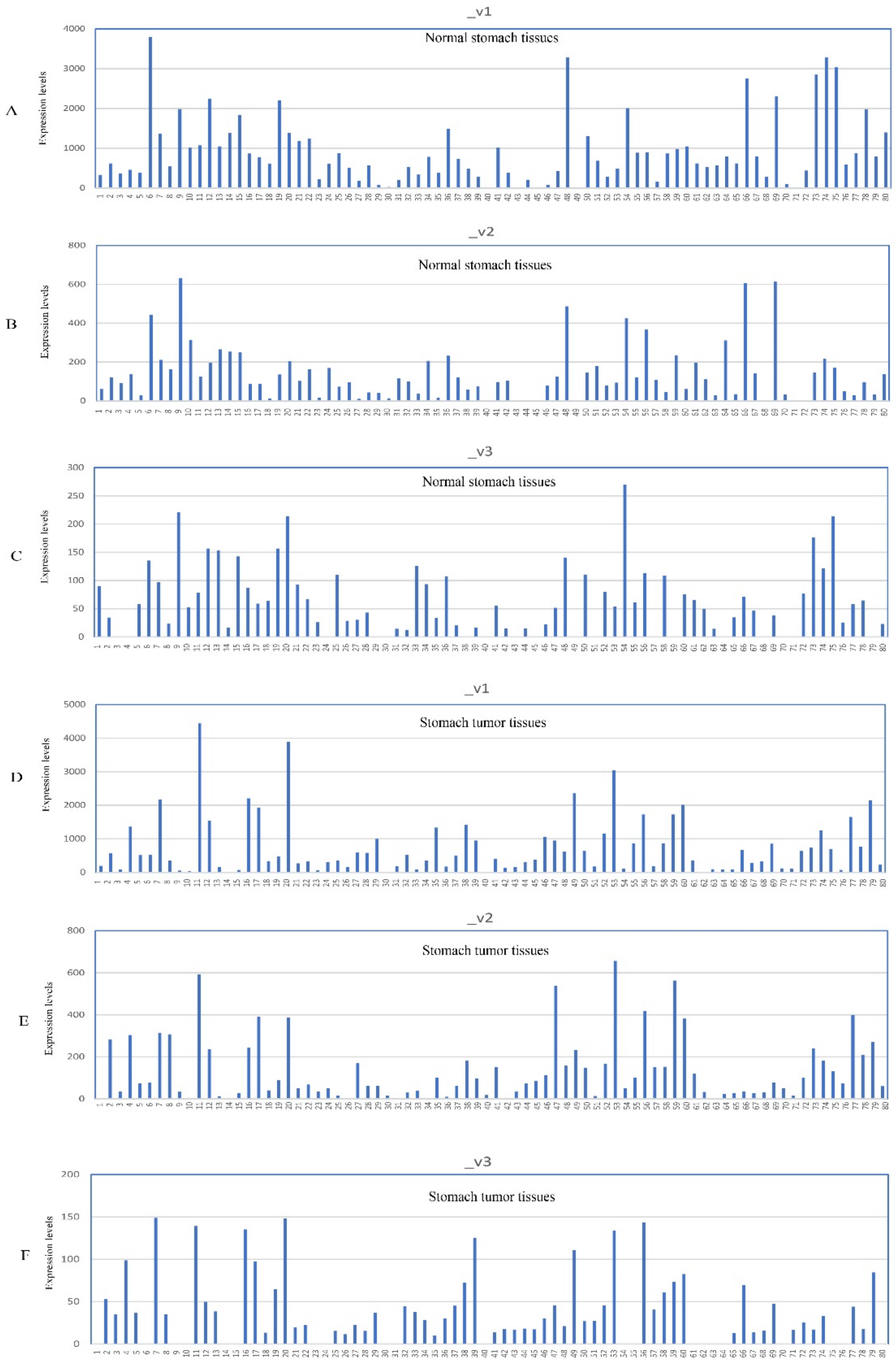

Figure S6. Expression profiles of UGT1A transcripts in stomach cancer and matched adjacent normal stomach tissues. The RNA-seq dataset of 80 paired stomach cancer and adjacent normal stomach tissues was downloaded the NCBI database and the sequence reads of UGT1A transcripts (_v1, _v2, _v3) were obtained using the SRA toolkit. Shown are the expression levels of UGT1A_v1, _v2, and _v3 transcripts in normal liver tissues (A-C) and RCC tumors (D-F).

A

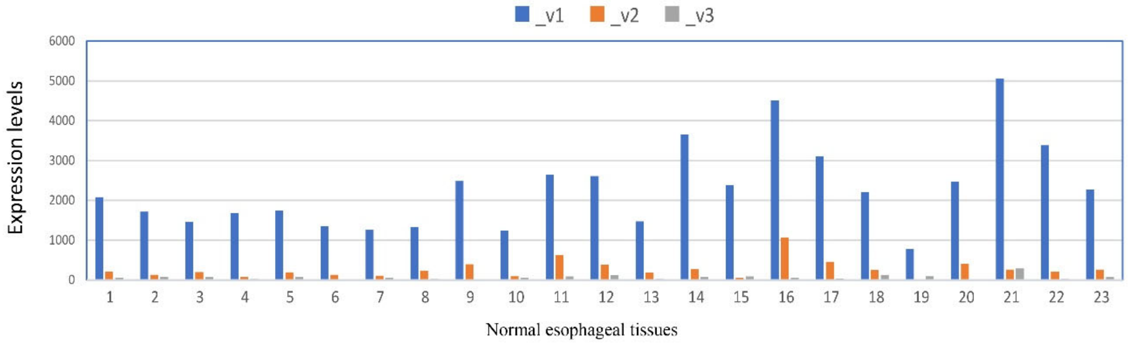

B

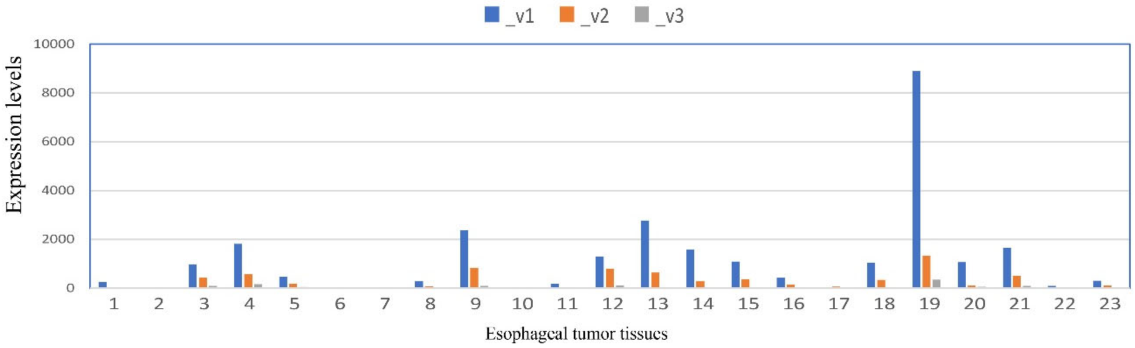

Figure S7. Expression profiles of UGT1A transcripts in esophagus cancer and matched adjacent normal esophagus tissues. The RNA-seq dataset of 23 paired esophagus cancer and adjacent normal esophagus tissues was downloaded the NCBI database and the sequence reads of UGT1A transcripts (_v1, _v2, _v3) were obtained using the SRA toolkit. Shown are the expression levels of UGT1A_v1, _v2, and _v3 transcripts in normal (A) and tumor (B) esophagus tissues.

A

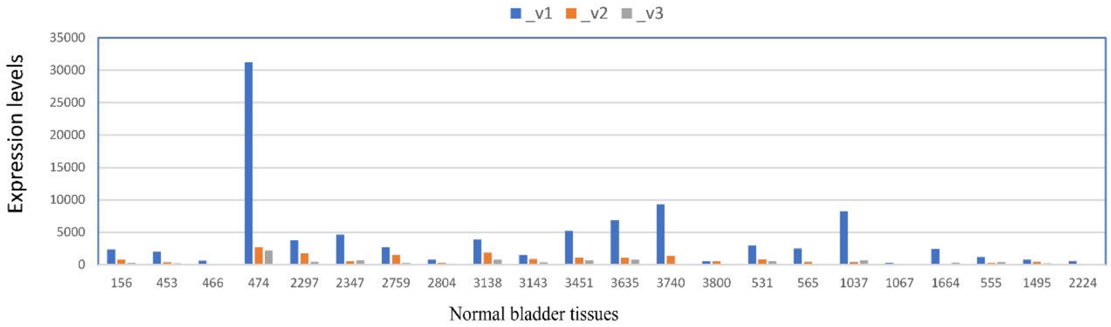

B

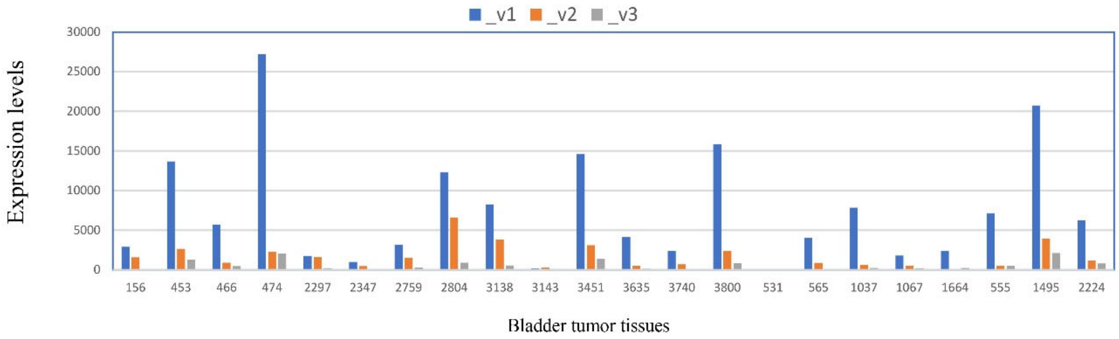

Figure S8. Expression profiles of UGT1A transcripts in bladder cancer and matched adjacent normal bladder tissues. The RNA-seq dataset of 22 paired bladder cancer and adjacent normal bladder tissues was downloaded the NCBI database and the sequence reads of UGT1A transcripts (_v1, _v2, _v3) were obtained using the SRA toolkit. Shown are the expression levels of UGT1A_v1, _v2, and _v3 transcripts in normal (A) and tumor (B) bladder tissues.

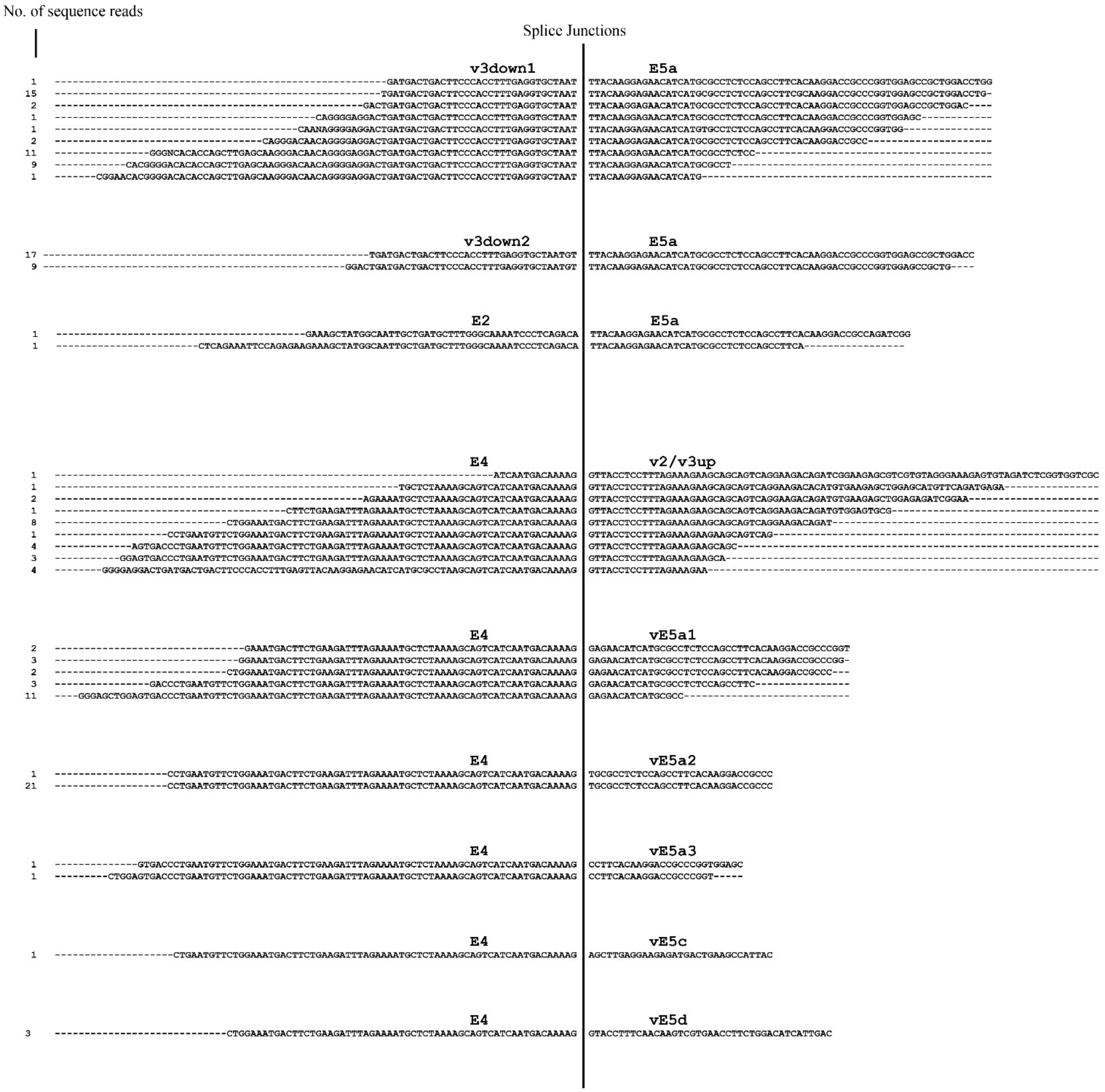

Figure S9. Sequence reads containing specific splice junctions of novel UGT1A transcripts. The UGT-captureSeq data (SRP073607) was downloaded from the NCBI database and the sequence reads containing specific splice junctions of novel UGT1A transcripts were obtained using the SRA toolkit. Shown are the sequence reads containing specific splice junctions of nine different novel UGT1A transcripts (as indicated) that were identified from UGT-CaptureSeq samples.

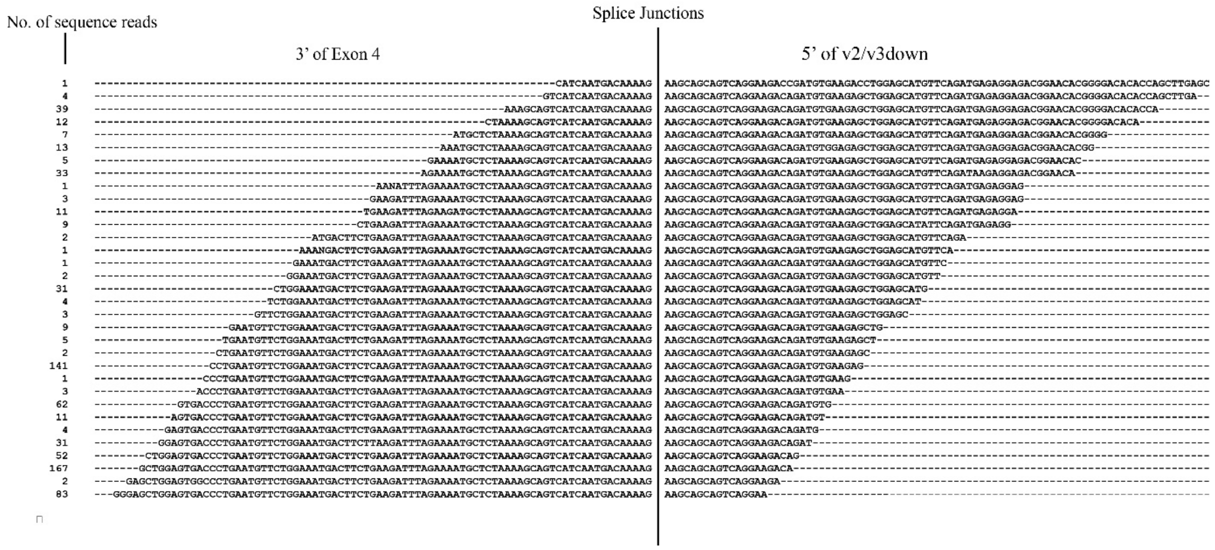

Figure S10. Sequence reads containing the specific splice junction of novel transcript v2/v3down. The UGT-captureSeq data (SRP073607) was downloaded from the NCBI database and the sequence reads containing the specific splice junction of UGT1A transcripts v2/v3down were obtained using the SRA toolkit. Shown are the sequence reads containing the v2/v3down-specific splice junction that were identified from UGT-CaptureSeq samples.

Table S13. UGT1A transcripts and proteins

Transcripts	Predicted Exon structure	Proteins	C-terminal peptides encoded by wildtype and variant 3' terminal exons	
			Lengths (aa)	Predicted Amino acid sequences
_v1	E1/E2/E3/E4/E5a	_i1	99	SYKENIMRLS SLHKDRPVEPLDLAVFWVEFVMRHKGAPHLRPAAHDLTWYQYHSLDVIGFLLAVVLTVAFITFKCCAYGYRKCLGKKGRVKKAHKSKTH
_v2	E1/E2/E3/E4/E5b	_i2	10	RKKQQSGRQM
_v3	E1/E2/E3/E4/E5bv/E5a	_i2	10	RKKQQSGRQM
_v2/v3up	E1/E2/E3/E4/EEv2/v3up	_iv2/v3up	44	RLPPLERSSSQEDRCEELEHVQMRGDGTRGHTSLSKGQQGRTDD
_v2/v3down	E1/E2/E3/E4/Ev2/v3down	_iv2/v3down	39	RSSSQEDRCEELEHVQMRGDGTRGHTSLSKGQQGRTDD
_vE5a1	E1/E2/E3/E4/E5a1	_iE5a1	28	RRTSCASPAFTRTARWSRWTWPCSGWSL
_vE5a2	E1/E2/E3/E4/E5a2	_iE5a2	67	SAPLQPSQGPPGGAAGPGRVLGGVCDEAQGRATPAPRSPRPHLVPVPFLGRDWFPLGRRADSGLHHL
_vE5a3	E1/E2/E3/E4/E5a3	_iE5a3	89	SLHKDRPVEPLDLAVFWVEFVMRHKGAPHLRPAAHDLTWYQYHSLDVIGFLLAVVLTVAFITFKCCAYGYRKCLGKKGRVKKAHKSKTH
_vE5c	E1/E2/E3/E4/E5c	_iE5c	2	RA
_vE5d	E1/E2/E3/E4/E5d	_iE5d	13	RYLSTSREPSGHH
_vE2E5a	E1/E2/E5a	_iE2E5a	72	LQGEHHAPLQPSQGPPGGAAGPGRVLGGVCDEAQGRATPAPRSPRPHLVPVPFLGRDWFPLGRRADSGLHHL

Amino acid sequences highlighted in RED (_i1, _iE5a3), BLUE (_iv2/v3up, _v2/v3down), or GREEN (_iE5a2, _iE2E5a) are encoded by the same Open reading frame. E: exon; aa: amino acid
